# Mechanisms associated with pyrethroid resistance in populations of *Aedes aegypti* (Diptera: Culicidae) from the Caribbean coast of Colombia

**DOI:** 10.1371/journal.pone.0228695

**Published:** 2020-10-06

**Authors:** Paula X. Pareja-Loaiza, Liliana Santacoloma Varon, Gabriela Rey Vega, Doris Gómez-Camargo, Ronald Maestre-Serrano, Audrey Lenhart

**Affiliations:** 1 Estudiante, Doctorados Nacionales Colciencias, Grupo UNIMOL, Doctorado en Medicina Tropical, Facultad de Medicina, Universidad de Cartagena, Cartagena de Indias, Colombia; 2 Laboratorio de Entomologia, Subdireccion Laboratorio Nacional de Referencia, Direccion Redes en Salud Publica, Instituto Nacional de Salud, Bogotá, Colombia; 3 Grupo UNIMOL, Doctorado en Medicina Tropical, Facultad de Medicina, Universidad de Cartagena, Cartagena de Indias, Colombia; 4 Facultad de Ciencias de la Salud, Universidad Simón Bolivar, Barranquilla, Colombia; 5 Centers for Disease Control and Prevention, Atlanta, Georgia, United States of America; Faculty of Science, Ain Shams University (ASU), EGYPT

## Abstract

*Aedes aegypti* is the main vector of dengue, chikungunya, and Zika viruses, which are of great public health importance in Colombia. *Aedes* control strategies in Colombia rely heavily on the use of organophosphate and pyrethroid insecticides, providing constant selection pressure and the emergence of resistant populations. In recent years, insecticide use has increased due to the increased incidence of dengue and recent introductions of chikungunya and Zika. In the present study, pyrethroid resistance was studied across six populations of *Ae*. *aegypti* from the Caribbean coast of Colombia. Susceptibility to λ-cyhalothrin, deltamethrin, and permethrin was assessed, and resistance intensity was determined. Activity levels of enzymes associated with resistance were measured, and the frequencies of three *kdr* alleles (V1016I, F1534C, V410L) were calculated. Results showed variations in pyrethroid susceptibility across *Ae*. *aegypti* populations and altered enzyme activity levels were detected. The *kdr* alleles were detected in all populations, with high variations in frequencies: V1016I (frequency ranging from 0.15–0.70), F1534C (range 0.94–1.00), and V410L (range 0.05–0.72). In assays of phenotyped individuals, associations were observed between the presence of V1016I, F1534C, and V410L alleles and resistance to the evaluated pyrethroids, as well as between the VI_1016_/CC_1534_/VL_410_ tri-locus genotype and λ-cyhalothrin and permethrin resistance. The results of the present study contribute to the knowledge of the mechanisms underlying the resistance to key pyrethroids used to control *Ae*. *aegypti* along the Caribbean coast of Colombia.

## Introduction

*Aedes aegypti (Stegomyia aegypti)* (Linnaeus, 1762) is the main vector of the dengue (DENV), chikungunya (CHIKV), and Zika (ZIKV) viruses. The diseases caused by these viruses are of growing public health importance worldwide owing to increased proliferation of mosquito populations, increased urbanization, as well as climatic and other environmental conditions suitable for transmission [1].

Globally, the burden of disease caused by dengue is increasing; it is estimated that approximately 390 million dengue infections occur each year, of which 96 million manifest clinically [2]. In 2015, 2.35 million cases of dengue were reported in the Americas, of which >10,200 cases were diagnosed as severe dengue, causing 1,181 deaths. In Colombia, dengue is considered a public health priority owing to its endemic transmission as well as the increased occurrence of severe dengue outbreaks, simultaneous circulation of all four DENV serotypes, and the occurrence of epidemic cycles every 2–3 years. In Colombia, the largest dengue epidemic was recorded in 2010, with >150,000 confirmed cases and 217 deaths [3]. Moreover, during 2007–2017, 609,228 cases of dengue were reported, of which 119,888 (19.7%) occurred in the Caribbean Region, specifically in the departments of Atlántico, Cesar, Córdoba, Sucre, Bolívar, La Guajira, Magdalena, and San Andrés y Providencia [4].

Chikungunya and Zika viruses were recently introduced into Colombia, in 2014 and 2015, respectively [5, 6]. The prevention and control activities of these three *Aedes*-borne arboviruses in Colombia have focused mainly on their principal vector, *Ae*. *aegypti*, through educational campaigns aimed at the community for the elimination of breeding sites, the application of biological and chemical insecticides, and the use of growth [7, 8]. The constant selection pressure arising from the heavy use of pyrethroid and organophosphate insecticides has generated resistant populations of *Ae*. *aegypti* in multiple areas of Colombia [9–15].

Resistance to insecticides in mosquitoes can be caused by the following mechanisms: behavioral modifications resulting in lessened likelihood of exposure, decreased penetration of the insecticide across the mosquito cuticle, alterations occurring at the insecticide target site within the mosquito, and increased detoxification (also referred to as metabolic resistance); the latter two mechanisms are the most frequently studied [16]. Target site alterations are most commonly caused by *kdr* mutations on the voltage-dependent sodium channel gene *para*, which is the target site for pyrethroids and DDT, or by mutations on the *Ace-1* gene (coding for the enzyme acetylcholinesterase), which is the target site for organophosphate and carbamate insecticides [17]. Metabolic resistance arises due to the increased activity or expression of genes coding for the main detoxifying enzymes including glutathione S-transferases, mixed-function oxidases, and esterases [16].

In Colombia, the insecticide susceptibility status of *Ae*. *aegypti* populations has been monitored for more than a decade. Since 2004, the National Insecticide Resistance Surveillance Network, headed by Colombia’s National Institute of Health, has evaluated approximately 170 populations of *Ae*. *aegypti* in 26 of the 32 departments in Colombia. The findings demonstrate variability in susceptibility to the insecticides temephos, λ-cyhalothrin, deltamethrin, permethrin, cyfluthrin, etofenprox, malathion, fenitrothion, pirimiphos-methyl, bendiocarb, and propoxur [12–29]. Moreover, increased activity levels of insecticide-degrading enzymes, such as nonspecific esterases, mixed-function oxidases (MFOs), glutathione S-transferases (GSTs), and insensitive acetylcholinesterase (iAChE), have been observed in resistant populations [9–13, 24]. In addition, the *kdr* mutations V1016I [13, 30], F1534C [31], and V410L [15] associated with pyrethroid resistance have recently been detected.

Specifically, in the Caribbean region, populations of *Ae*. *aegypti* have been reported with variable levels of susceptibility to organophosphates, resistance to pyrethroids, and alterations in the enzymatic activity of glutathione-S-transferases, alpha-esterases and mixed function oxidases [13]. Additionally, the *kdr* mutations V1016I [13, 30], F1534C [31, 32] and V410L [15] have been detected.

The present study builds upon earlier work by further investigating the intensity and spatial extent of pyrethroid resistance in *Ae*. *aegypti* along the Caribbean coast of Colombia and links the frequency of *kdr* alleles and tri-locus *kdr* haplotypes to insecticide resistant phenotypes. To further understand the mechanisms of resistance, we also analyzed the activity levels of key detoxification enzyme groups.

## Materials and methods

The insectary that was used to rear mosquitoes belonged to the Public Health Laboratory of the Department of Atlántico. Mosquitoes were reared using their existing protocols which include the routine use of feeding mosquito colonies on mice. This is standard practice in government insectaries in Colombia and is overseen by the Colombian National Institute of Health’s National Public Health Laboratory Network.

### *Ae. aegypti* collections

**Fig 1:**
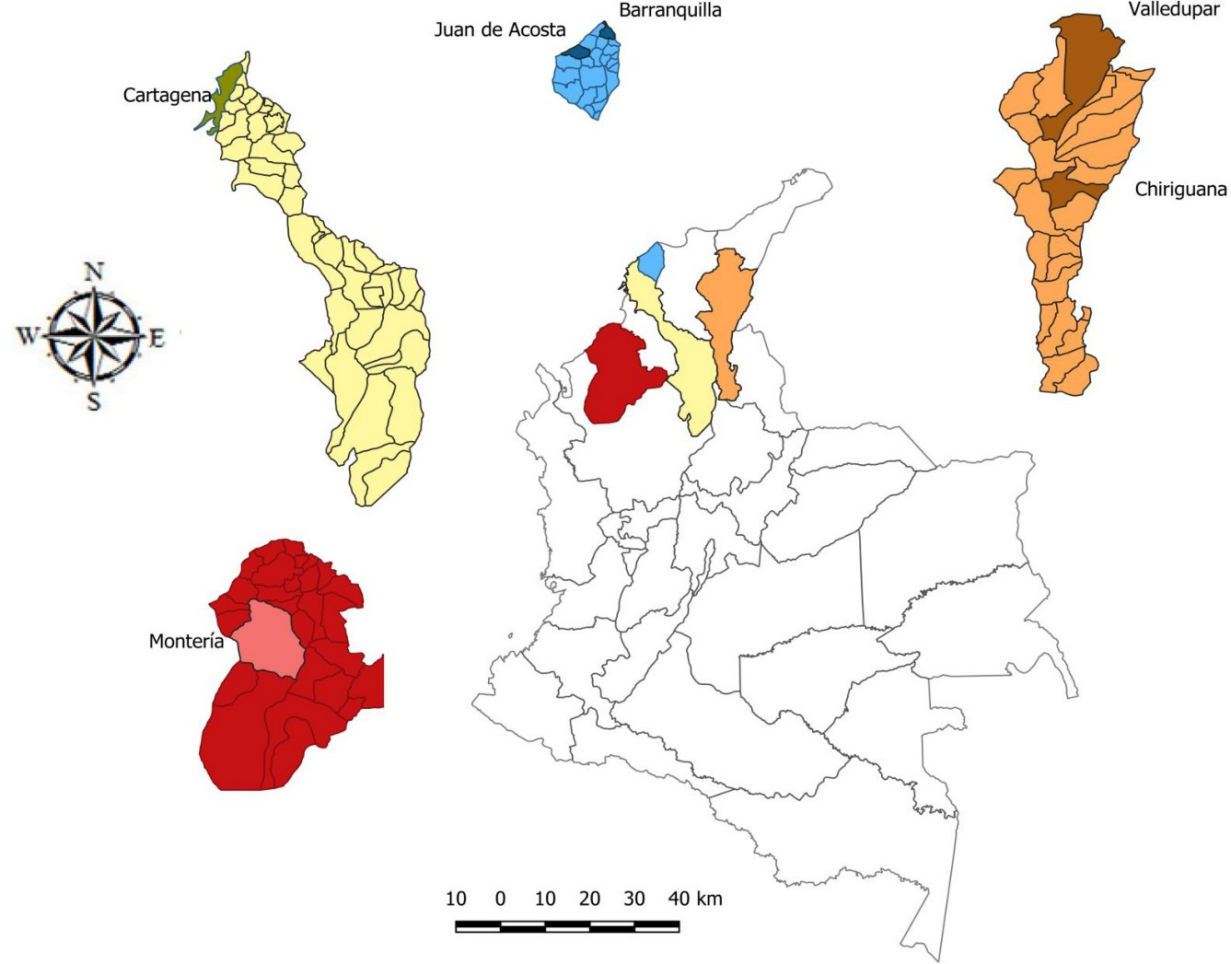
*Aedes aegypti* collection sites located in the Caribbean region of Colombia. Montería (red), Bolívar (yellow), Atlántico (blue) and Cesar (orange).

*Ae*. *aegypti* were collected in the municipalities of Barranquilla (N 10° 57' 10.622'', W 75° 49' 12.024'') and Juan de Acosta (N 10° 49' 44.731'', W 75° 2' 9.088'') in the department of Atlántico; Cartagena (N 10° 24' 55.416'', W 75° 27' 38.485'') in the department of Bolívar; Valledupar (N 9° 56' 55.068'', W 73° 38' 4.164'') and Chiriguaná (N 9° 21' 41.27'', W 73° 35' 58.919'') in the department of Cesar; and Montería (N 8° 44' 30.866'', W 75° 52' 0.433'') in the department of Córdoba (Fig 1).

The study was undertaken in collaboration with and under the supervision of the Entomology Laboratory of the Colombian National Institute of Health, which coordinates the Entomology Laboratory Network in Colombia. This network for entomological surveillance includes the monitoring of insecticide resistance in insects of public health importance, to guide decision-making in the control of vector-borne diseases at the national level. The project also had the support of the local health secretaries in each of the departments selected for sampling sites.

The study sites were selected taking into account the national insecticide susceptibility baseline records. Municipalities with a susceptibility baseline were selected in order to identify variations in susceptibility over time and expand knowledge regarding enzymatic and molecular resistance mechanisms. Sites without an existing baseline were also selected in order to generate baseline data regarding the susceptibility to insecticides in these areas.

Immature *Aedes* were collected from habitats including tanks, pools, plastic/metallic cans, tires, animal water dishes, and flower vases located around houses. The specimens were reared to adults and maintained under controlled conditions of temperature (28°C ± 2°C), relative humidity (60% ± 10%), and photoperiod (12 h light:12 h dark) in the Public Health Laboratory of the department of Atlántico.

Upon emergence, male mosquitoes were fed with 10% sugar solution, and the females were fed with mouse (*Mus musculus)* blood every third day to obtain eggs of the F1 generation. Eggs were stored in an airtight plastic container, until they were hatched to obtain the adult mosquitoes used in the bioassays.

### Bioassays

Insecticide bioassays were performed following the methodologies described by the CDC [33] and WHO [34]. The pyrethroid insecticides and their concentrations were as follows: λ-cyhalothrin (10 μg/bottle [CDC] and 0.03% treated papers WHO]), deltamethrin (10 μg/bottle [CDC] and 0.03% treated papers WHO]), and permethrin (15 μg/bottle [CDC] and 0.25% treated papers WHO]). The technical grade insecticides (Chem Service®) used for the CDC bioassays were provided by the National Insecticide Resistance Surveillance Network of the Colombian National Institute of Health. The insecticide-impregnated papers used for the WHO bioassays were provided by University Sains Malaysia.

For each population, 20–25 F1 generation, 3- to 5-day-old, unfed female *Ae*. *aegypti* were used in each bioassay replicate; as a control, the susceptible Rockefeller laboratory *Ae*. *aegypti* strain was used. Each bioassay consisted of four replicates per insecticide for each population. The diagnostic time post-exposure was 30 min for the CDC bioassays and 24h for the WHO bioassays. Upon the completion of the diagnostic time, the living and dead specimens were classified as phenotypically resistant (R) or susceptible (S), and individually stored in 0.5 mL tubes with a hole in the lid and desiccated in tightly sealed bags containing silica gel. The bags containing the tubes were stored at −80°C for the subsequent detection of the V1016I, F1534C, and V410L *kdr* alleles.

In populations where resistance was detected via the CDC bioassay, resistance intensity was determined by conducting additional bioassays employing 2X the original insecticide concentration [34].

### Biochemical assays

Biochemical assays were conducted on F1 generation adults. One day post-emergence, 40 unfed female *Ae*. *aegypti* from each population were preserved at −80°C until processing. Individuals from the susceptible Rockefeller strain were used as controls. Mosquitoes were homogenized individually in 30 μL of distilled water for 5–10 seconds with an electric macerator and an additional 270 μL of distilled water was added for a final volume of 300 μL. Subsequently, samples were centrifuged at 12,000 rpm for 60 seconds and aliquoted in triplicate in 96 well microplates: 10 μL for α, β, pNPA-esterases; 15 μL for GST; 20 μL for mixed-function oxidases (MFO); and 25 μL for iAChE. For the tests of MFO and acetylcholinesterase, the samples were transferred without being centrifuged. Enzyme activity levels were determined according to the methodology described by Valle *et al*. [35], which measures the optical densities at predetermined wavelengths to estimate the activity levels of MFO, iAChE, esterases, and GSTs. Total protein concentration was also determined for each individual mosquito to correct for differences in body sizes [36]. Results were read using an ELISA plate reader (Multiskan^TM^-Thermo Fisher Scientific®).

### Detection of *kdr* alleles

Real-time PCR was used to identify the V1016I, F1534C, and V410L *kdr* mutations. To estimate the allele frequencies in natural populations, 40–50 *Ae*. *aegypti* parental (F0) mosquitoes from each population were analyzed. To estimate associations between genotype and phenotype, all phenotypically resistant (R) and 30 randomly selected susceptible (S) individuals were analyzed per insecticide per population.

DNA was extracted from individual mosquitoes using the Quanta Biosciences Extracta^TM^ Kit. Individual mosquitoes were placed in sterile 0.2 mL tubes and 25 μL extraction buffer was added to each tube, followed by an incubation at 95°C for 30 min in a C1000 Bio-Rad CFX 96 Touch^TM^ Real-Time System thermocycler. At the end of the incubation, 25 μL of stabilization buffer was added. DNA was quantified using a NanoDrop^TM^ 2000/2000c spectrophotometer (ThermoFisher Scientific).

PCR reactions were performed in a Bio-Rad C1000 CFX96 Real-Time System thermocycler. Genotype was determined by analyzing the melting curves of the PCR products. The V1016I mutation was amplified following the methodology described by Saavedra-Rodríguez *et al*. [37], using a final reaction volume of 20 μL, containing 6 μL of ddH_2_O, 10 μL of iQ^TM^ SYBR® Green Supermix (Bio-Rad), 1 μL of each of the V1016f, I1016f, and I1016r primers (Table 1), and 1 μL of DNA template. The cycling conditions were as follows: an initial denaturation at 95°C for 3 min followed by 40 cycles of: 95°C for 10 s, 60°C for 10 s, and 72°C for 30 s; and a final extension at 95°C for 10 s. The melting curves were determined by a denaturation gradient from 65°C to 95°C with an increase of 0.2°C every 10 seconds.

**Table 1:** Primer sequences used for detecting *kdr* alleles.

Mutation	Primer	Sequence (5´–3´)
V1016I	V1016(f)	5´-CGGGCAGGGCGGCGGGGGCGGGGCCACAAATTGTTTCCCACCCGCACCGG-3´
	I1016(f)	5´-GCGGGCACAATTGTTTCCCACCCGCACTGA-3´
	I1016(r)	5´-GGATGAACCGAAATTGGACAAAAGC-3´
F1534C	C1534(f)	5´-GCGGGCAGGGCGGCG GGGGCGGGGCCTCTACTTTGTGTTCTTCATCATGTG-3´
	F1534(f)	5´-GCGGGCTCTACTTTGTGTTCTTCATCATATT-3´
	F1534(r)	5´-TCTGCTCGTTGAAGTTGTCGAT-3´
V410L	V410(f)	5´-GCGGGCAGGGCGGCGGGGGCGGGGCCATCTTCTTGGGTTCGTTCTACCGTG-3´
	L410(f)	5´-GCGGGCATCTTCTTGGGTTCGTTCTACCATT-3´
	L410(r)	5´-TTCTTCCTCGGCGGCCTCTT-3´

The F1534C mutation was detected following the methodology described by Yanola *et al*. [38], using a final reaction volume of 20 μL comprised of 7.15 μL of ddH_2_O, 9 μL of iQ^TM^ SYBR® Green Supermix (Bio-Rad), 0.6 μL of each of the F1534f, C1534f, and F1534r primers (Table 1), 0.65 μL of the C1534f primers, and 2 μL of DNA template. The cycling conditions were as follows: an initial denaturation at 95°C for 3 min followed by 37 cycles of: 95°C for 10 s, 57°C for 30 s, and 72°C for 30 s; and a final extension at 95°C for 10 s. The melting curves were determined by a denaturation gradient from 65°C to 95°C with an increase of 0.5°C every 5 s.

The V410L mutation was detected following the methodology described by Haddi *et al*. [39], using a final reaction volume of 21 μL comprised of 9.6 μL of ddH_2_O, 10 μL of iQ^TM^ SYBR® Green Supermix (Bio-Rad), 0.1 μL of each of the L410f, V410f, and L410r primers (Table 1), 0.2 μL of the L410r primer, and 1.0 μL of DNA template. The cycling conditions were as follows: an initial denaturation at 95°C for 3 min followed by 39 cycles of: 95°C for 10 s, 60°C for 10 s, and 72°C for 30 s; and a final extension at 95°C for 10 s. The melting curves were determined by a denaturation gradient from 65°C to 95°C with an increase of 0.2°C every 10 s.

Each mosquito was analyzed in duplicate. For all assays for each mutation, three positive controls were included: a wild-type homozygote, a homozygote mutant, and a heterozygote. All assays also included a negative control consisting of master mix without DNA template.

### Data analysis

#### Bioassays

Mortality was scored at the diagnostic time per insecticide per population. Populations were categorized according to the WHO criteria [34], whereby 98%–100% mortality indicates susceptibility, 90%–97% suggests resistance is developing and <90% mortality indicates resistance.

#### Biochemical assays

Absorbance values were entered into Excel databases to calculate the mean and standard deviation for each mosquito. To express the absorbance values in terms of enzymatic activity, data regarding the homogenate volume of each mosquito, total protein content for each mosquito, and units of activity for each enzyme group were calculated according to the protocol described by Valle *et al*. [35]. The cutoff value for the susceptible Rockefeller strain was determined based on the 99^th^ percentile of absorbance, and the percentage of individuals from the field strains with activity levels that exceeded this cutoff value were classified according to the criteria proposed by Montella *et al*. [40]: <15% unaltered, 15%–50% altered, and >50% highly altered.

After determining the activity levels for each enzyme group, an analysis of variance was performed, followed by Tukey’s multiple comparison test, with the significance level set at *p* ≤ 0.05, to identify populations with any statistically significant differences as compared to the Rockefeller reference strain.

#### Allelic and genotypic frequencies of the V1016I, F1534C, and V410L mutations

Results were obtained using Bio-Rad's Precision Melt Analysis Software^TM^ and were interpreted as follows. For the V1016I mutation, a melting peak at 77°C corresponded to a mutant homozygote (I/I), a peak at 82°C corresponded to a wild-type homozygote (V/V), and peaks at both 77°C and 82°C corresponded to a heterozygote (V/I). For the F1534C mutation, a peak at 82°C corresponded to a mutant homozygote (C/C), a peak at 78°C corresponded to a wild-type homozygote (F/F), and peaks at both 78°C and 82°C corresponded to a heterozygote (F/C). For the V410L mutation, a peak at 80°C corresponded to a mutant homozygote (L/L), a peak at 83°C corresponded to a wild-type homozygote (V/V), and peaks at both 80°C and 83°C corresponded to a heterozygote (V/L).

From the parental mosquitoes (F0), the population-level allele frequencies for I1016, C1534, and L410 were calculated using Eq (1) as follows

$$\frac{n\;heterozygotes+2\;(n\;homozygotes)}{2\;(total\;n\;mosquitoes\;analyzed)}$$ (1)

The genotypic frequencies for V_1016_/V_1016_, F_1534_/F_1534_, V_410_/V_410_, I_1016_/I_1016_, C_1534_/C_1534_, L_410_/L_410_, V_1016_/I_1016_, F_1534_/C_1534_, V_410_/L_410_ were calculated using Eq (2).

$$\frac{n\;mosquitoes\;with\;the\;genotype\;to\;be\;calculated}{total\;n\;mosquitoes\;analyzed}$$ (2)

The Hardy–Weinberg principle was tested, as shown in Eq (3)

$$p^{2}+2pq+q^{2}=1$$ (3)

where p is the number of wild-type homozygotes, pq is the frequency of heterozygotes, and q is the frequency of mutant homozygotes.

Expected wild-type V_1016_/V_1016_, F_1534_/F_1534_, V_410_/V_410_ homozygotes = p^2^ (n).

Expected V_1016_/I_1016_, F_1534_/C_1534_, V_410_/L_410_ heterozygotes = 2pq (n).

Expected mutant I_1016_/I_1016_, C_1534_/C_1534_, L_410_/L_410_ homozygotes = q^2^ (n).

The Chi square test was used to determine whether the populations were in Hardy–Weinberg equilibrium, as shown in Eq (4):

$$x^{2}calc=\sum \frac{{\left(f_{0}-f_{e}\right)}^{2}}{f_{e}}$$ (4)

f_0: Frecuency observed value_.

f_e: Frecuency expected value_.

If the calculated value of χ^2^ was < tabulated χ^2^ (1 gl) = 3.84 and P < 0.05, the H_0_ that the study population was in Hardy–Weinberg equilibrium was accepted; otherwise, if the calculated χ^2^ was ≥ tabulated χ^2^, the H_a_ that the study population was not in Hardy–Weinberg equilibrium was accepted.

In addition, the coefficient of endogamy was calculated using Eq (5) as follows:

$$F_{IS}=1-\left(\frac{H_{obs}}{H_{exp}}\right)$$ (5)

where, H_obs_ is the number of observed heterozygotes and H_exp_ is the number of expected heterozygotes; if F_IS_ was significantly higher than 0, an excess of homozygotes was considered, and if F_IS_ was significantly less than 0, an excess of heterozygotes was considered in the population, with a significance of P < 0.05. In addition, the frequencies of tri-locus genotypes were determined in the study populations.

#### Association of *kdr* mutations with pyrethroid resistance

The association between resistant and susceptible phenotypes and their *kdr* genotypes was tested using contingency tables, and the relationship between phenotype and tri-locus genotype was tested using the statistical software programs OpenEpi version 3.0 (https://www.openepi.com/TwobyTwo/TwobyTwo.htm) and GraphPad Prism version 8.1.

## Results

### Bioassays

**Fig 2:**
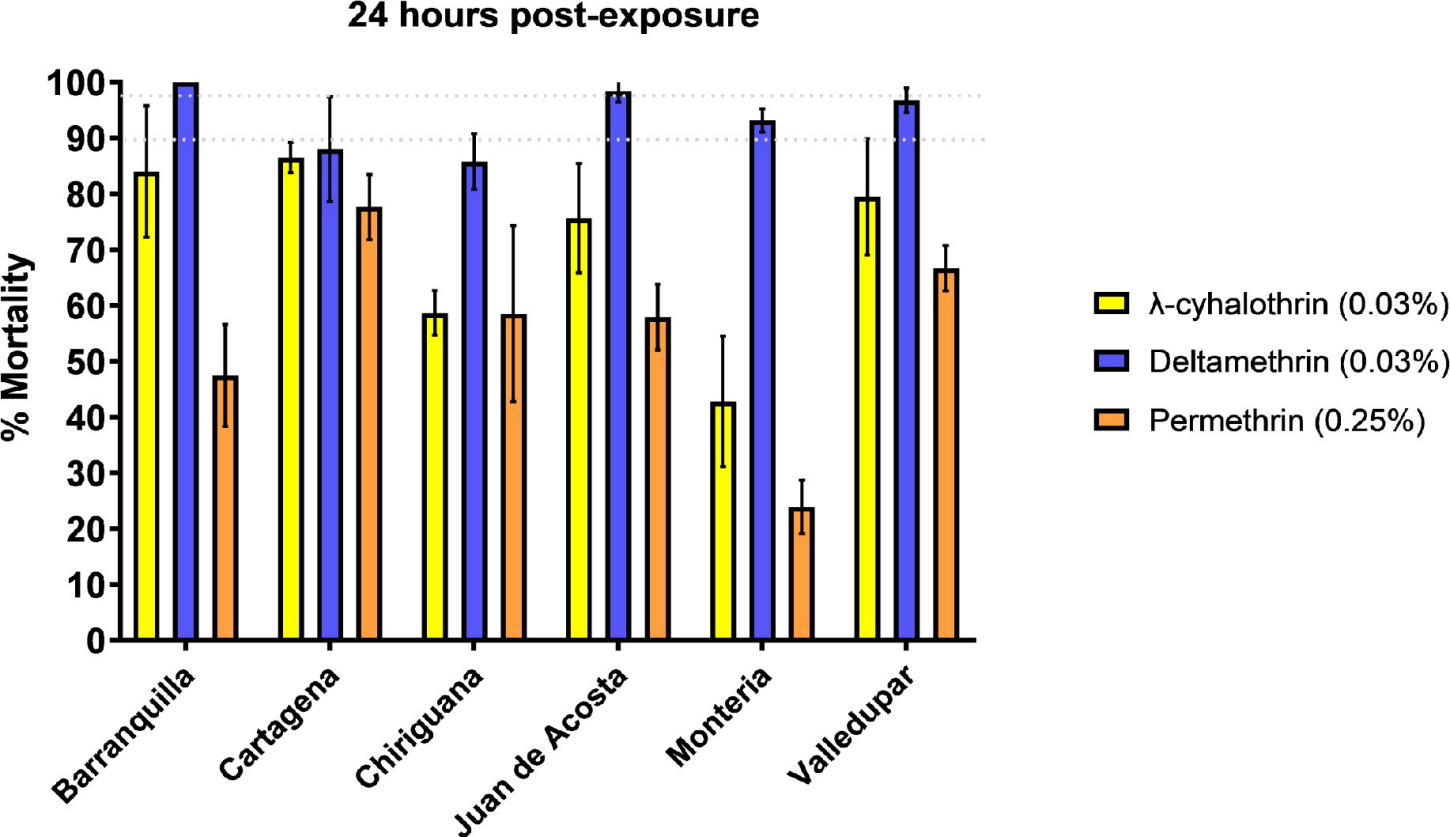
Mortality of the six populations of *Ae*. *aegypti* evaluated against diagnostic concentrations of pyrethroid insecticides following WHO bioassay methodology.

A total of 1732 adult female *Ae*. *aegypti* were tested in WHO bioassays for susceptibility to λ-cyhalothrin (n = 564), deltamethrin (n = 586), and permethrin (n = 582). Resistance to λ-cyhalothrin and permethrin was detected in all six evaluated populations. Resistance was most frequent in Montería with 43.3% mortality to λ-cyhalothrin and 24.0% mortality to permethrin. Cartagena was the least resistant, with mortalities of 86.4% to λ-cyhalothrin and 77.6% to permethrin. Susceptibility to deltamethrin was observed in the populations from Juan de Acosta (98% mortality) and Barranquilla (100% mortality), and possible development of resistance was detected in Valledupar (96.8% mortality) and Montería (93.2% mortality). The populations from Cartagena (87.9% mortality) and Chiriguaná (86.0% mortality) were found to be resistant to deltamethrin (Fig 2).

**Fig 3:**
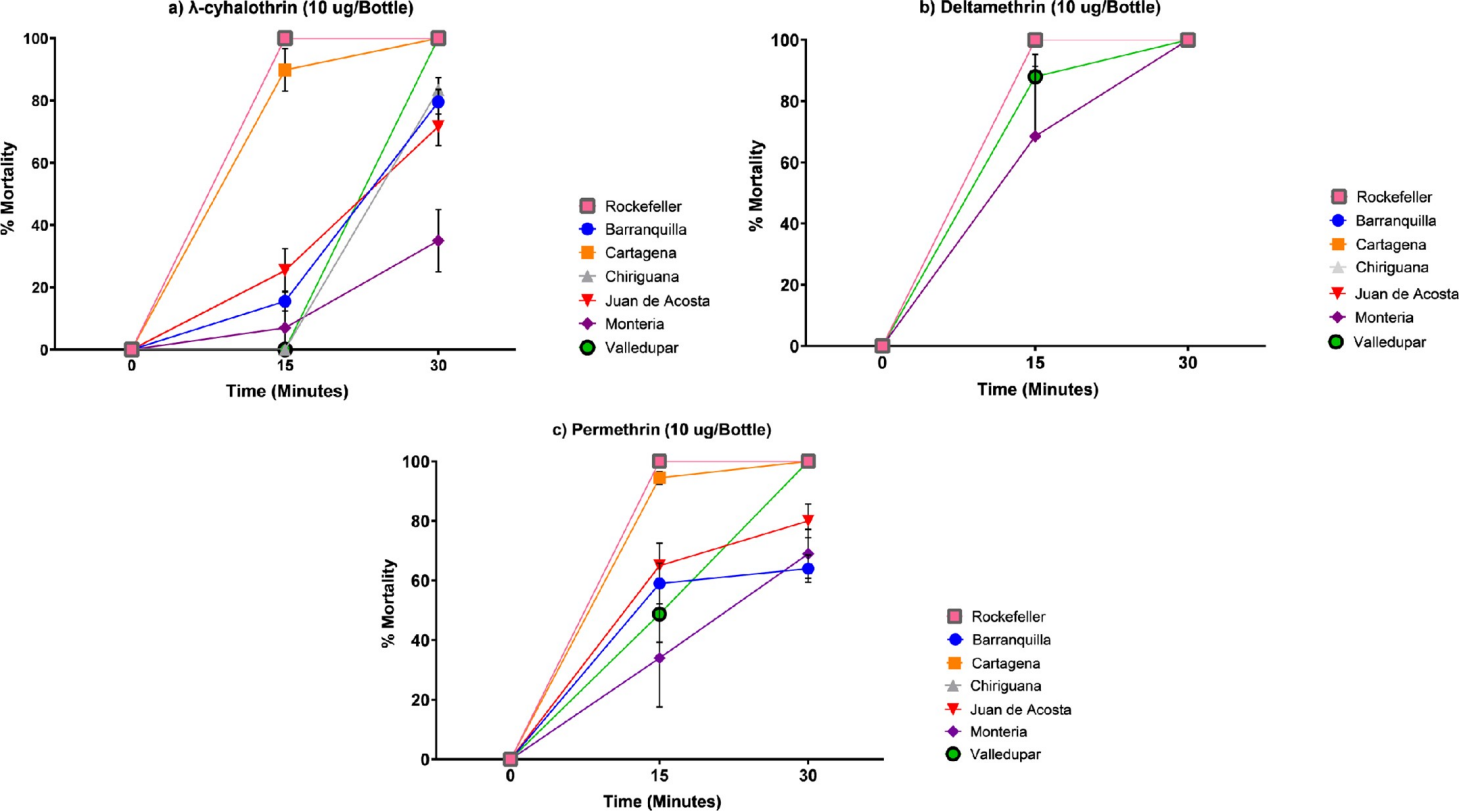
Mortality of the six populations of *Ae*. *aegypti* evaluated against diagnostic doses of pyrethroid insecticides following CDC bioassay methodology. a) λ-cyhalothrin (10ug/bottle), b) Deltamethrin (10ug/bottle, c) Permethrin (15ug/bottle).

Additionally, a total of 1822 adult female *Ae*. *aegypti* were tested in CDC bioassays for susceptibility to λ-cyhalothrin (n = 606), deltamethrin (n = 608), and permethrin (n = 608). Resistance to λ-cyhalothrin was detected in the populations from Barranquilla (79.6% mortality), Chiriguaná (83.5% mortality), Juan de Acosta (71.6% mortality), and Montería (35% mortality), whereas the populations from Cartagena (98.0% mortality) and Valledupar (100% mortality) were susceptible (Fig 3A). Susceptibility to deltamethrin was observed in all populations, with mortalities of 100% (Fig 3B). Resistance to permethrin was detected in the populations from Juan de Acosta (80.0% mortality), Montería (69.0% mortality), and Barranquilla (64% mortality), and susceptibility was observed in the populations from Cartagena, Chiriguaná and Valledupar, with mortalities of 100% (Fig 3C). (Fig 3).

In populations where resistance intensity was assessed, 100% mortality at the diagnostic time was observed after exposure to twice the concentration (2X) of the recommended diagnostic dose for λ-cyhalothrin (20 μg/bottle) and permethrin (30 μg/bottle) (Table 2).

**Table 2:** Mortality of *Ae*. *aegypti* exposed to 1X and 2X the diagnostic doses of λ-cyhalothrin and permethrin.

**Insecticide**	**Populations**	**1× DD**		**2× DD**	
		**10 μg/bottle**		**20 μg/bottle**	
		**n**^**a**^	**Mortality (%)**	**n**	**Mortality (%)**
**λ-cyhalothrin** (DD^b^: 10 μg/bottle, DT^c^: 30 min)	Barranquilla	103	76.61	100	100
	Chiriguaná	103	83.49	100	100
	Juan de Acosta	102	71.56	100	100
	Montería	100	35.0	100	100
**Insecticide**	**Populations**	**15 μg/bottle**		**30 μg/bottle**	
		**N**	**Mortality (%)**	**n**	**Mortality (%)**
**Permethrin** (DD: 15 μg/bottle, DT: 30 min)	Barranquilla	100	64.0	100	100
	Juan de Acosta	100	80.0	100	100
	Montería	100	69.0	100	100

^a^ Total number of females evaluated.

^b^ Diagnostic dose.

^c^ Diagnostic time.

### Biochemical assays

**Fig 4:**
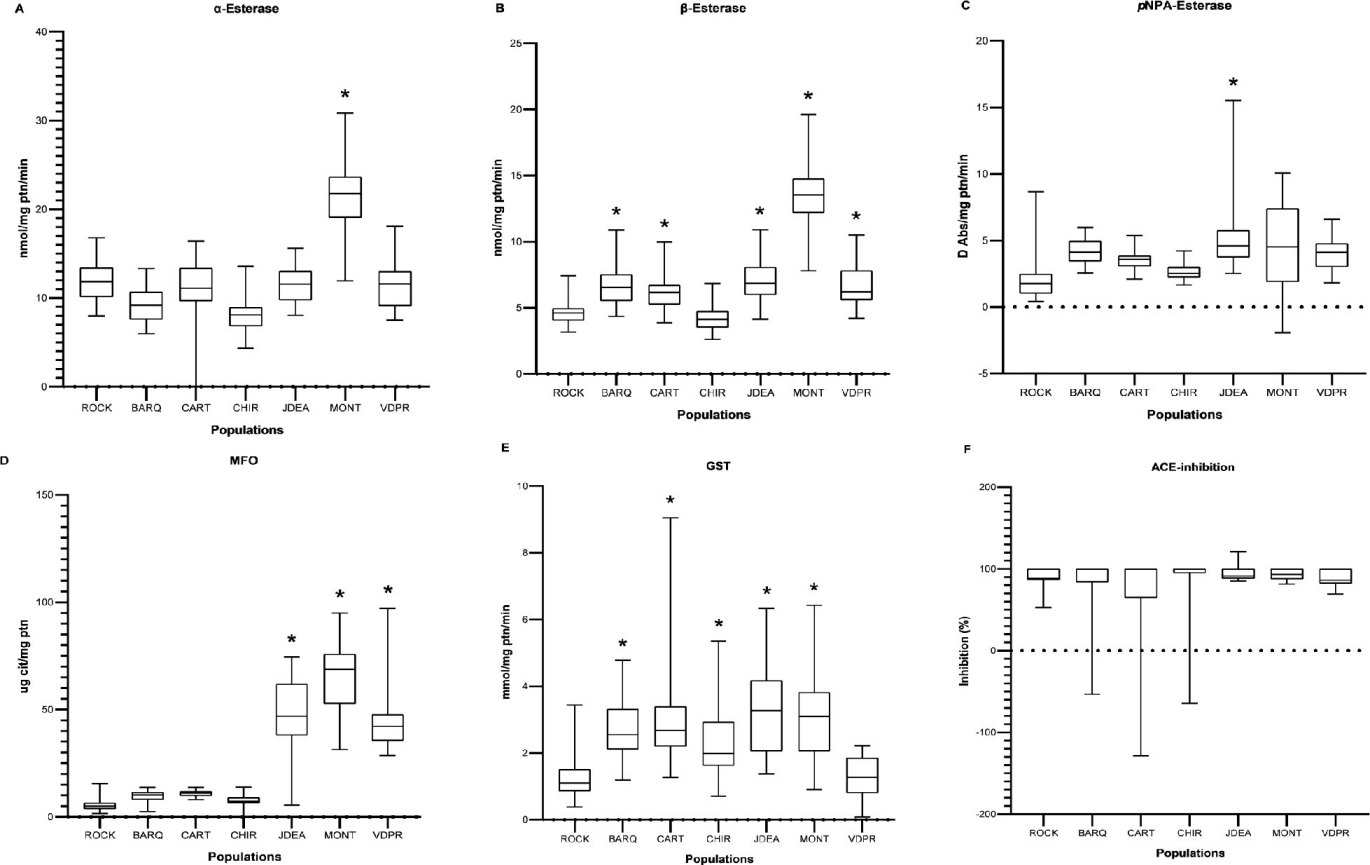
Box plots of enzymatic activity levels. *Aedes aegypti* populations with elevated enzymatic activity compared to the Rockefeller strain are marked with (*). (A) α-esterases, (B) β-esterases, (C) pNPA- esterases, (D) mixed-function oxidases (MFO), (E) glutathione-S-transferases (GSTs), and (F) insensitive acetylcholinesterase (iAChE). ROCK: Rockefeller; BARQ: Barranquilla-, CART: Cartagena; CHIR: Chiriguaná; JDEA: Juan de Acosta; MONT: Montería and VDPR: Valledupar.

Based on the classification criteria of Montella *et al*. [40], α-esterase enzyme levels were highly altered in the population from Montería, where 79% of individuals exceeded the 99^th^ percentile of the Rockefeller reference population (Fig 4A). Similarly, β-esterase activity levels were highly altered in the population of Montería (97%) and were also altered in the populations from Juan de Acosta (45%), Barranquilla (31%), Valledupar (27%) and Cartagena (12%) (Fig 4B), and pNPA-esterase activity levels were altered in the population from Juan de Acosta (14%) (Fig 4C). Highly altered MFO activity levels were detected in the populations from Juan de Acosta (92%), Montería (97%), and Valledupar (88%) (Fig 4D). Altered GST activity levels were detected in the populations from Barranquilla (17%), Cartagena (24%), Juan de Acosta (44%), Montería (34%) and Chiriguaná (4%) (Fig 4E). AChE activity remained unaltered in all populations evaluated (Fig 4F). Overall, significant differences were observed between the mean activity levels of most enzyme groups between the field populations and the Rockefeller reference strain (p < 0.05) (Fig 4).

### *kdr* allele frequencies

All three *kdr* mutations were detected in all the populations evaluated. Regarding the V1016I mutation, all three genotypes (VV_1016_, VI_1016_, and II_1016_) were detected in each field population. The mutant allele I1016 was the most prevalent in the population from Montería, with a frequency of 0.70, and the least prevalent in the populations from Barranquilla and Valledupar, with a frequency of 0.15 in both. Regarding the F1534C mutation, all three genotypes (FF_1534_, FC_1534_, and CC_1534_) were detected in the populations from Barranquilla and Juan de Acosta, whereas only FC_1534_ and CC_1534_ were detected in Cartagena, Chiriguaná, and Valledupar, with CC_1534_ predominating in all populations. It is noteworthy that the CC_1534_ genotype was fixed in the population from Montería with a frequency of 1.0 (Table 3). In addition, the frequency of the C1534 mutant allele in the populations from Cartagena, Valledupar, and Chiriguaná ranged between 0.94 and 0.97, but was 0.76 in the populations from Barranquilla and Juan de Acosta. Regarding the V410L mutation, all three genotypes (VV_410_, VL_410_, and LL_410_) were detected in each field population. The highest frequency of the L410 allele was detected in Montería with a frequency of 0.72, whereas the lowest was detected in Valledupar with a frequency of 0.05. For the other populations, the frequencies of the L410 allele ranged between 0.12 and 0.32 (Table 3).

**Table 3:** Genotype and allele frequencies of the V1016I, F1534C, and V410L *kdr* mutations in F0 *Ae*. *aegypti* females.

**Population**	**n**^**a**^	**Genotype frequency**			**Allele frequency**		**Hardy–Weinberg**		**F**_**IS**_
		**V1016I**			**V1016I**				
		**VV**	**VI**	**II**	**V**	**I**	**χ2**	***p* value**	
Barranquilla	49	0.71	0.27	0.02	0.85	0.15	0.02	0.87	-0.02
Cartagena	46	0.74	0.20	0.07	0.84	0.16	3.68	0.06	0.28
Chiriguaná	47	0.57	0.34	0.09	0.74	0.26	0.51	0.47	0.10
Juan de Acosta	48	0.75	0.23	0.04	0.86	0.16	0.88	0.34	0.15
Montería	43	0.09	0.42	0.49	0.30	0.70	0.00	0.95	0.00
Valledupar	48	0.73	0.25	0.02	0.85	0.15	0.00	0.98	−0.00
		**F1534C**			**F1534C**		**χ2**	***p* value**	**F**_**IS**_
	**N**	**FF**	**FC**	**CC**	**F**	**C**			
Barranquilla	49	0.10	0.29	0.61	0.24	0.76	2.53	0.11	0.22
Cartagena	46	0.00	0.07	0.93	0.03	0.97	0.05	0.82	−0.03
Chiriguaná	47	0.00	0.11	0.89	0.05	0.95	0.15	0.70	−0.05
Juan de Acosta	48	0.04	0.44	0.54	0.26	0.76	0.80	0.37	−0.10
Montería	43	0.00	0.00	1.00	0.00	1.00	-	-	-
Valledupar	48	0.00	0.13	0.87	0.06	0.94	0.21	0.64	−0.07
		**V410L**			**V410L**		**χ2**	***p* value**	**F**_**IS**_
	**N**	**VV**	**VL**	**LL**	**V**	**L**			
Barranquilla	49	0.02	0.20	0.78	0.88	0.12	0.12	0.72	0.05
Cartagena	46	0.07	0.37	0.57	0.75	0.25	0.00	0.92	0.01
Chiriguaná	47	0.09	0.47	0.45	0.68	0.32	0.28	0.59	−0.07
Juan de Acosta	48	0.02	0.27	0.71	0.84	0.16	0.03	0.85	−0.02
Montería	43	0.51	0.42	0.07	0.28	0.72	0.07	0.79	−0.04
Valledupar	48	0.04	0.02	0.94	0.95	0.05	29.88	0.00	0.79

^a^ Number of mosquitoes evaluated.

F_IS_ inbreeding coefficient.

For loci 1016 and 1534, all genotypes were found to be in Hardy–Weinberg equilibrium. In the case of locus 410, the genotypes of most populations, except Valledupar, were in Hardy–Weinberg equilibrium (p < 0.05). When determining the inbreeding coefficients (F_IS_) for I1016, values < 0 were obtained for the populations from Barranquilla and Valledupar due to an excess of heterozygotes, in contrast to the populations from Cartagena, Chiriguaná, Juan de Acosta, and Montería, where values > 0 were recorded due to a deficiency of heterozygotes. For C1534, a generalized excess of heterozygotes was observed, with the exception of Barranquilla, where a deficiency of heterozygotes was observed. Similarly, for L410, the populations from Barranquilla, Cartagena, and Valledupar showed a deficiency of heterozygotes, in contrast to Chiriguaná, Juan de Acosta, and Montería, where an excess of heterozygotes was detected (Table 3).

**Fig 5:**
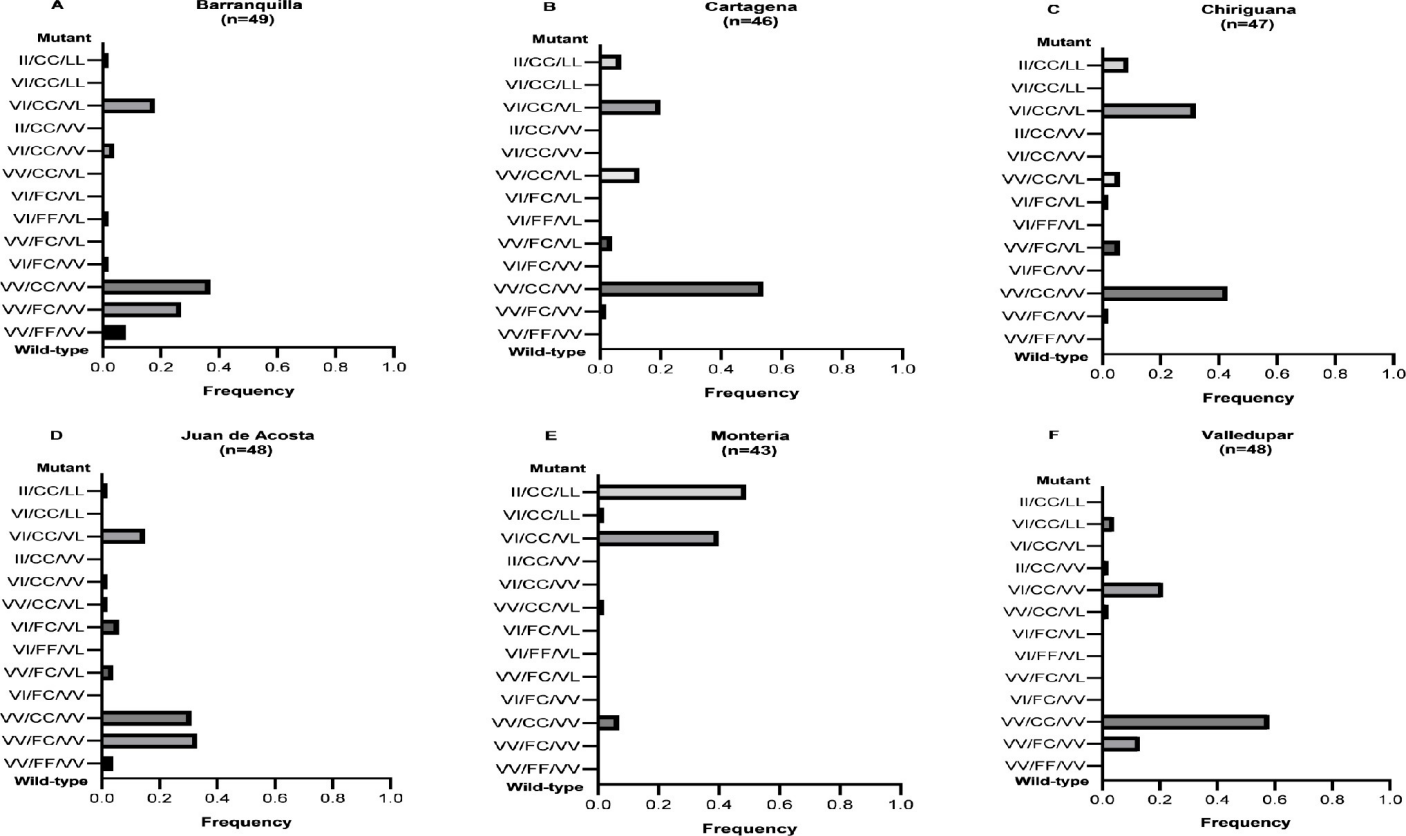
Frequencies of the 13 tri-locus genotypes present in F0 *Ae*. *aegypti* females. The order of the genotypes is 1016/1534/410. Mutant alleles: 1016 = I, 1534 = C, and 410 = L. The triple-mutant homozygous genotype is shown at the top and the triple-wild-type homozygous genotype at the bottom of each chart.

Of the 27 combinations of tri-locus genotypes, 13 combinations were detected in 281 mosquitoes collected from the six evaluated populations. The triple homozygous wild-type genotype (VV_1016_/ FF_1534_/VV_410_) was detected only in the populations from Barranquilla and Juan de Acosta, with frequencies of 0.08 and 0.04, respectively, whereas the triple homozygous mutant genotype (II_1016_/CC_1534_/LL_410_) was present in all populations except Valledupar, with frequencies between 0.02 (Barranquilla) and 0.49 (Montería). Similarly, the triple heterozygous genotype (VI_1016_/FC_1534_/VL_410_) was present only in Chiriguaná and Juan de Acosta at low frequencies (0.02 and 0.06, respectively). The homozygous wild-type genotype for loci 1016 and 410 and homozygous resistant for locus 1534 (VV_1016_/CC_1534_/VV_410_) was most frequent in Barranquilla, Cartagena, Chiriguaná, and Valledupar, with frequencies of 0.37, 0.54, 0.43, and 0.58, respectively; the exceptions were Juan de Acosta, where the most frequent genotype was homozygous wild-type for loci 1016 and 410 and heterozygous for locus 1534 (VV_1016_/FC_1534_/VV_410_) with a frequency of 0.33, and Montería, where the most frequent genotype was the triple homozygous mutant (II_1016_/CC_1534_/LL_410_), with a frequency of 0.49 (Fig 5).

### Association of *kdr* alleles with phenotypic resistance to pyrethroids

Based on the results obtained with the mosquitoes exposed to insecticides in the WHO bioassays, a significant association (p < 0.05) was identified between the mutant *kdr* alleles 1016I, 1534C, and 410L and resistance to λ-cyhalothrin in the populations from Juan de Acosta, Montería, and Valledupar. Similarly, an association was observed between the 1534C allele and resistance to deltamethrin in the populations of Chiriguaná, Montería, and Valledupar and between the 1016I and 410L alleles and resistance to deltamethrin in the population of Montería. A significant association (p < 0.05) was also detected between the 1016I, 1534C, and 410L alleles and resistance to permethrin in the populations from Chiriguaná, Montería, and Valledupar; between the 1534C allele and resistance to permethrin in Barranquilla, Cartagena, and Juan de Acosta; and between the 410L allele and permethrin resistance in Juan de Acosta (Tables 4–6).

**Table 4:** Association between 1016I, 1534C, and 410L alleles and resistance to λ-cyhalothrin in adult *Ae*. *aegypti* in WHO bioassays.

λ-cyhalothrin							
	*kdr* mutation	Genotype	n^a^	Phenotype		OR^d^ (95%CI)^e^	*p* value^f^
				R^b^	S^c^		
**Barranquilla**		**II**	0	0	0		
	**V1016I**	**VI**	15	4	11	0.80 (0.23–2.83)	0.740
	** **	**VV**	28	9	19		
		**CC**	22	6	16		
	**F1534C**	**FC**	18	5	13	0.63 (0.23–1.70)	0.361
	** **	**FF**	3	2	1		
		**LL**	0	0	0		
	**V410L**	**VL**	12	3	9	0.74 (0.18–2.99)	0.670
		**VV**	31	10	21		
**Cartagena**		**II**	2	1	1		
	**V1016I**	**VI**	9	2	7	1.26 (0.34–4.59)	0.726
	** **	**VV**	30	8	22		
		**CC**	30	10	20		
	**F1534C**	**FC**	8	0	8	2.5 (0.512–12.2)	0.245
	** **	**FF**	3	1	2		
		**LL**	2	1	1		
	**V410L**	**VL**	8	2	6	1.44 (0.39–5.38)	0.582
		**VV**	31	8	33		
**Chiriguaná**		**II**	12	6	6		
	**V1016I**	**VI**	22	13	9	0.50 (0.23–1.06)	0.069
	** **	**VV**	28	21	7		
		**CC**	53	37	16		
	**F1534C**	**FC**	9	3	6	4.05 (0.96–17.09)	0.042
	** **	**FF**	0	0	0		
		**LL**	9	6	3		
	**V410L**	**VL**	21	13	8	0.97 (0.44–2.15)	0.948
	** **	**VV**	32	21	11		
**Juan de Acosta**		**II**	3	3	0		
	**V1016I**	**VI**	26	15	11	3.02 (1.28–7.11)	*0.009
	** **	**VV**	27	8	19		** **
	** **	**CC**	35	22	13		
	**F1534C**	**FC**	20	4	16	5.14 (1.61–16.40)	*0.003
	** **	**FF**	1	0	1		
		**LL**	4	3	1		** **
	**V410L**	**VL**	23	14	9	2.78 (1.19–6.58)	*0.017
		**VV**	29	9	20		** **
**Montería**		**II**	16	12	4		
	**V1016I**	**VI**	37	33	4	4.85 (2.31–10.18)	*0.000
	** **	**VV**	29	7	22		
		**CC**	63	47	16		
	**F1534C**	**FC**	15	4	11	6.46 (2.38–17.51)	*0.000
	** **	**FF**	4	1	3		
		**LL**	11	11	0		
	**V410L**	**VL**	36	28	8	6.02 (2.60–13.91)	*0.000
	** **	**VV**	35	13	22		
**Valledupar**		**II**	1	1	0		
	**V1016I**	**VI**	5	4	1	13.62 (1.56–118.80)	*0.003
	** **	**VV**	40	11	29		
		**CC**	12	11	1		
	**F1534C**	**FC**	23	5	18	10.8 (3.61–32.28)	*0.000
	** **	**FF**	11	0	11		
		**LL**	1	1	0		
	**V410L**	**VL**	5	4	1	13.62 (1.56–118.8)	*0.003
	** **	**VV**	40	11	29		

^a^Sample size

^b^Resistant mosquitoes

^c^Susceptible mosquitoes

^d^Odds ratio for the association between the mutant alleles 1016I, 1534C, and 410L and resistance to λ-cyhalothrin

^e^Lower and upper limits of the confidence interval for the OR

^f^Significant difference (p < 0.05)–Pearson X^2^.

**Table 5:** Association between 1016I, 1534C, and 410L alleles and resistance to deltamethrin in adult *Ae*. *aegypti* in WHO bioassays.

Deltamethrin							
	*kdr* mutation	Genotype	n^a^	Phenotype		OR^d^ (95%CI)^e^	*p* value^f^
				R^b^	S^c^		
**Cartagena**		**II**	1	0	1		
	**V1016I**	**VI**	15	3	12	0.52 (0.13–2.01)	0.337
	** **	**VV**	25	8	17		
	** **	**CC**	22	4	18		
	**F1534C**	**FC**	15	6	9	0.58 (0.20–1.66)	0.312
	** **	**FF**	4	1	3		
		**LL**	1	0	1		
	**V410L**	**VL**	15	3	12	0.52 (0.13–2.01)	0.337
		**VV**	25	8	17		
**Chiriguaná**		**II**	0	0	0		
	**V1016I**	**VI**	9	4	5	3.14 (0.74–13.26)	0.105
	** **	**VV**	30	5	25		
		**CC**	11	6	5		
	**F1534C**	**FC**	21	3	18	5.71 (1.50–21.81)	*0.006
	** **	**FF**	7	0	7		
		**LL**	0	0	0		
	**V410L**	**VL**	6	3	3	3.8 (0.70–20.77)	0.103
		**VV**	33	6	27		
**Juan de Acosta**		**II**	0	1	0		
	**V1016I**	**VI**	16	1	15	9 (0.90–93.17)	0.031
	** **	**VV**	15	0	15		** **
		**CC**	20	2	18		
	**F1534C**	**FC**	9	0	9	0.0	0.253
		**FF**	3	0	3		
		**LL**	1	1	0		
	**V410L**	**VL**	14	0	14	3.29 (0.42–25.50)	0.233
		**VV**	17	1	16		
**Montería**		**II**	5	4	1		
	**V1016I**	**VI**	16	4	12	6.57 (2.08–20.71)	*0.000
	** **	**VV**	18	1	17		
		**CC**	29	9	20		
	**F1534C**	**FC**	8	0	8	0.0	*0.039
	** **	**FF**	2	0	2		
		**LL**	5	4	1		** **
	**V410L**	**VL**	18	4	14	5.5 (1.77–17.11)	*0.001
		**VV**	16	1	15		
**Valledupar**		**II**	0	0	0		
	**V1016I**	**VI**	3	0	3	0.0	0.517
	** **	**VV**	31	4	27		
		**CC**	5	3	2		
	**F1534C**	**FC**	16	0	16	6 (1.11–32.45)	*0.022
	** **	**FF**	13	1	12		
		**LL**	0	0	0		
	**V410L**	**VL**	3	0	3	0.0	0.517
		**VV**	31	4	27		

^a^Sample size

^b^Resistant mosquitoes

^c^Susceptible mosquitoes

^d^Odds ratio for the association between the mutant alleles 1016I, 1534C, and 410L and resistance to deltamethrin

^e^Lower and upper limits of the confidence interval for the OR

^f^Significant difference (p < 0.05)–Pearson X^2^.

**Table 6:** Association between 1016I, 1534C, and 410L alleles and resistance to permethrin in adult *Ae*. *aegypti* in WHO bioassays.

Permethrin							
	*kdr* mutation	Genotype	n^a^	Phenotype		OR^d^ (95%CI)^e^	*p* value^f^
				R^b^	S^c^		
**Barranquilla**		**II**	3	3	0		
	**V1016I**	**VI**	18	11	7	1.78 (0.66–4.78)	0.249
	** **	**VV**	30	17	13		
		**CC**	35	22	13		
	**F1534C**	**FC**	20	8	12	3.01 (1.28–7.09)	*0.009
	** **	**FF**	6	1	5		
		**LL**	1	1	0		
	**V410L**	**VL**	19	12	7	2.21 (0.82–5.93)	0.110
	** **	**VV**	41	18	23		
**Cartagena**		**II**	2	1	1		
	**V1016I**	**VI**	19	10	9	0.57 (0.62–3.98)	0.337
	** **	**VV**	32	12	20		
		**CC**	32	17	15		
	**F1534C**	**FC**	17	6	11	3.09 (1.12–8.53)	*0.025
	** **	**FF**	4	0	4		
		**LL**	2	1	1		
	**V410L**	**VL**	18	9	9	1.4 (0.55–3.59)	0.482
	** **	**VV**	33	13	20		
**Chiriguaná**		**II**	3	3	0		
	**V1016I**	**VI**	20	18	2	13.92 (3.13–61.84)	*0.000
	** **	**VV**	44	16	28		
		**CC**	21	18	3		
	**F1534C**	**FC**	33	18	15	5.01 (2.40–10.50)	*0.000
	** **	**FF**	13	1	12		
		**LL**	4	4	0		
	**V410L**	**VL**	17	16	1	28.32 (3.70–216.80)	*0.000
	** **	**VV**	46	17	29		
**Juan de Acosta**		**II**	13	8	5		
	**V1016I**	**VI**	26	17	9	1.58 (0.78–3.20)	0.201
	** **	**VV**	30	14	16		
		**CC**	48	32	16		
	**F1534C**	**FC**	20	7	13	3.38 (1.27–8.93)	*0.010
	** **	**FF**	1	0	1		
		**LL**	7	6	1		
	**V410L**	**VL**	30	19	11	2.38 (1.11–5.12)	*0.023
	** **	**VV**	32	14	18		** **
**Montería**		**II**	23	20	3		
	**V1016I**	**VI**	29	24	5	2.99 (1.40–6.35)	*0.003
	** **	**VV**	40	24	16		
		**CC**	70	64	6		
	**F1534C**	**FC**	16	4	12	33 (10.51–103.60)	*0.000
	** **	**FF**	6	0	6		
		**LL**	21	20	1		
	**V410L**	**VL**	25	21	4	5.69 (2.27–14.28)	*0.000
	** **	**VV**	46	27	19		
**Valledupar**		**II**	3	3	0		
	**V1016I**	**VI**	19	16	3	10.45 (2.93–37.29)	*0.000
	** **	**VV**	39	12	27		
		**CC**	24	22	2		
	**F1534C**	**FC**	25	9	16	11.78 (4.85–28.60)	*0.000
	** **	**FF**	12	0	12		
		**LL**	3	3	0		
	**V410L**	**VL**	19	16	3	10.45 (2.93–37.29)	*0.000
	** **	**VV**	39	12	27		** **

^a^Sample size

^b^Resistant mosquitoes

^c^Susceptible mosquitoes

^d^Odds ratio for the association between the mutant alleles 1016I, 1534C, and 410L and resistance to permethrin

^e^Lower and upper limits of the confidence interval for the OR

^f^Significant difference (p < 0.05).

Less association was detected between *kdr* alleles and the observed phenotype in the CDC bioassays. A significant association (p < 0.05) between the 1534C allele and resistance to λ-cyhalothrin was detected in the population from Barranquilla and between the 1016I and 410L alleles and resistance to permethrin in the population from Montería. Despite the resistance to pyrethroids detected with the CDC bioassays in the populations from Chiriguaná and Juan de Acosta, no significant associations were detected between *kdr* alleles and resistant phenotypes in these populations (Tables 7 and 8).

**Table 7:** Association between 1016I, 1534C, and 410L alleles and resistance to λ-cyhalothrin in adult *Ae*. *aegypti* in CDC bioassays.

λ-cyhalothrin							
	*kdr* mutation	Genotype	n^a^	Phenotype		OR^d^ (95%CI)^e^	*p* value^f^
				R^b^	S^c^		
**Barranquilla**		**II**	0	0	0		
	**V1016I**	**VI**	16	9	7	2.06 (0.70–0.18)	0.182
	** **	**VV**	35	12	23		
	** **	**CC**	27	16	11		
	**F1534C**	**FC**	21	5	16	4.28 (1.47–12.51)	*0.005
	** **	**FF**	3	0	3		
		**LL**	0	0	0		
	**V410L**	**VL**	16	9	7	2.06 (0.70–6.07)	0.182
		**VV**	35	12	23		
**Chiriguaná**		**II**	6	3	3		
	**V1016I**	**VI**	22	7	15	1.15 (0.48–2.75)	0.753
	** **	**VV**	19	7	12		
		**CC**	43	16	27		
	**F1534C**	**FC**	4	1	3	1.73 (0.17–17.38)	0.634
	** **	**FF**	0	0	0		
		**LL**	6	3	3		
	**V410L**	**VL**	24	9	15	1.47 (0.62–3.46)	0.382
	** **	**VV**	17	5	12		
**Juan de Acosta**		**II**	4	3	1		
	**V1016I**	**VI**	21	11	10	1.94 (0.82–4.58)	0.126
	** **	**VV**	31	12	19		
		**CC**	29	14	15		
	**F1534C**	**FC**	23	10	13	1.07 (0.47–2.46)	0.867
	** **	**FF**	4	2	2		
		**LL**	4	2	2		
	**V410L**	**VL**	24	12	12	1.22 (0.54–2.78)	0.631
	** **	**VV**	28	12	16		
**Montería**		**II**	14	10	4		
	**V1016I**	**VI**	51	35	16	1.41 (0.77–2.57)	0.269
	** **	**VV**	35	20	15		
		**CC**	98	63	35		
	**F1534C**	**FC**	1	1	0	0.30 (0.01–6.12)	0.409
	** **	**FF**	1	1	0		
		**LL**	15	10	5		
	**V410L**	**VL**	50	34	16	1.20 (0.66–2.18)	0.545
		**VV**	35	21	14		

^a^Sample size

^b^Resistant mosquitoes

^c^Susceptible mosquitoes

^d^Odds ratio for the association between the mutant alleles 1016I, 1534C, and 410L and resistance to λ-cyhalothrin

^e^Lower and upper limits of the confidence interval for the OR

^f^Significant difference (p < 0.05).

**Table 8:** Association between 1016I, 1534C, and 410L alleles and resistance to permethrin in adult *Ae*. *aegypti* in CDC bioassays.

Permethrin							
	*kdr* mutation	Genotype	n^a^	Phenotype		OR^d^ (95%CI)^e^	*p* value^f^
				R^b^	S^c^		
**Barranquilla**		**II**	4	3	1		
	**V1016I**	**VI**	36	14	22	1.67 (0.84–3.29)	0.139
	** **	**VV**	60	29	41		
	** **	**CC**	54	21	33		
	**F1534C**	**FC**	35	10	25	1.05 (0.55–2.01)	0.865
	** **	**FF**	11	5	6		
		**LL**	3	2	1		
	**V410L**	**VL**	33	14	19	1.70 (0.83–3.45)	0.141
	** **	**VV**	64	20	44		
**Juan de Acosta**		**II**	2	1	1		
	**V1016I**	**VI**	21	9	12	1.25 (0.50–3.12)	0.637
	** **	**VV**	27	10	17		
		**CC**	24	9	15		
	**F1534C**	**FC**	24	9	15	0.69 (0.29–1.67)	0.413
	** **	**FF**	2	2	0		
		**LL**	2	1	1		
	**V410L**	**VL**	21	9	12	1.25 (0.50–3.12)	0.637
	** **	**VV**	27	10	17		
**Montería**		**II**	11	7	4		
	**V1016I**	**VI**	36	21	15	2.08 (1.01–4.30)	*0.045
	** **	**VV**	14	3	11		
	** **	**CC**	61	31	30		
	**F1534C**	**FC**	0	0	0	1.03 (0.02–52.91)	0.987
	** **	**FF**	0	0	0		
	** **	**LL**	11	7	4		
	**V410L**	**VL**	36	21	15	2.08 (1.01–4.30)	*0.045
	** **	**VV**	14	3	11		

^a^Sample size

^b^Resistant mosquitoes

^c^Susceptible mosquitoes

^d^Odds ratio for the association between the mutant alleles 1016I, 1534C, and 410L and resistance to λ-cyhalothrin

^e^Lower and upper limits of the confidence interval for the OR

^f^Significant difference (p < 0.05).

### Comparisons of tri-locus genotypes with resistance to pyrethroids

Of the 27 possible combinations of genotypes, 20 combinations of tri-locus genotypes were detected in the 918 mosquitoes phenotyped in WHO bioassays. The most common haplotypes were VV_1016_/CC_1534_/VV_410_ (n = 233 mosquitoes, 25.4%), VV_1016_/FC_1534_/VV_410_ (n = 198, 21.6%), and VI_1016_/CC_1534_/VL_410_ (n = 187, 20.4%). Wild-type double homozygotes at loci 1016 and 410 in the presence of CC1534/FC1534 were significantly more likely to be phenotypically susceptible to deltamethrin (p < 0.05). Heterozygotes at both loci 1016 and 410 in the presence of CC1534 were significantly more likely to be resistant to λ-cyhalothrin and permethrin (p < 0.05) and susceptible to deltamethrin (p < 0.05) (Table 9).

**Table 9:** Tri-locus genotypes of phenotyped adult *Ae*. *aegypti* from the six study populations after WHO bioassay.

			tri-locus genotype																						
Insecticide	Phenotype	n^c^	II/CC/LL	II/FC/LL	VI/CC/LL	II/CC/VL	VI/FC/LL	VV/CC/LL	II/FC/VL	VI/CC/VL	II/CC/VV	VI/CC/VV	VV/CC/VL	II/FC/VV	VI/FC/VL	VV/FF/LL	VI/FF/VL	VV/FC/VL	II/FF/VV	VI/FC/VV	VV/CC/VV	VV/FF/VL	VI/FF/VV	VV/FC/VV	VV/FF/VV
λ cyhalothrin	R^a^	158	22	0	0	1	0	0	0	56	0	7	0	0	7	0	0	0	0	0	47	0	1	14	3
	S^b^	172	4	0	0	5	1	0	0	18	2	6	0	0	18	0	0	0	0	0	47	0	0	53	18
Deltamethrin	R	35	5	0	0	0	0	0	0	8	0	1	0	0	2	0	0	0	0	1	10	0	0	6	2
	S	150	2	0	0	0	0	0	0	30	0	0	0	0	14	0	0	1	0	3	31	1	0	42	26
Permethrin	R	229	33	0	1	4	1	0	0	66	0	8	3	0	19	0	0	1	0	2	60	0	0	29	2
	S	174	2	0	1	3	0	0	1	9	0	2	0	1	21	0	1	0	1	2	38	0	0	54	38
Total		918	68	0	2	13	2	0	1	187	2	24	3	1	81	0	1	2	1	8	233	1	1	198	89

^a^Resistant (living)

^b^Susceptible (dead)

^c^Total number of mosquitoes. The order of the genotypes is shown for loci 1016/1534/410. Resistant allele at locus 1016 = I, 1534 = C, 410 = L, triple-resistant genotype II/CC/LL, triple-susceptible genotype VV/FF/VV. Significant differences between resistant and susceptible are shown in bold (p < 0.05).

From the CDC bioassays, 15 combinations of tri-locus genotypes were observed in 465 mosquitoes assayed with λ-cyhalothrin and permethrin in Barranquilla, Juan de Acosta, and Montería. Similar to the WHO bioassays, the most common haplotypes were VI_1016_/CC_1534_/VL_410_ (n = 161, 34.6%) and VV_1016_/CC_1534_/VV_410_ (n = 117, 25.2%). Wild-type double homozygotes at loci 1016 and 410 in the presence of CC1534/FC1534 were significantly more likely to be phenotypically susceptible to λ-cyhalothrin and permethrin (p < 0.05) (Table 10).

**Table 10:** Tri-locus genotypes of phenotyped adult *Ae*. *aegypti* after CDC bioassay.

			tri-locus genotype																						
Insecticide	Phenotype	n^c^	II/CC/LL	II/FC/LL	VI/CC/LL	II/CC/VL	VI/FC/LL	VV/CC/LL	II/FC/VL	VI/CC/VL	II/CC/VV	VI/CC/VV	VV/CC/VL	II/FC/VV	VI/FC/VL	VV/FF/LL	VI/FF/VL	VV/FC/VL	II/FF/VV	VI/FC/VV	VV/CC/VV	VV/FF/VL	VI/FF/VV	VV/FC/VV	VV/FF/VV
λ cyhalothrin	R^a^	129	14	0	1	2	0	0	0	52	0	2	3	0	5	0	1	1	0	1	35	0	0	10	2
	S^b^	125	7	0	1	1	0	2	0	41	0	1	1	0	5	0	0	2	0	0	33	0	0	25	6
Permethrin	R	87	10	0	0	1	0	0	0	34	0	1	0	0	9	0	0	0	0	0	15	0	0	10	7
	S	124	6	0	0	0	0	0	0	34	0	3	1	0	9	0	0	1	0	3	34	1	0	27	5
Total	** **	465	37	0	2	4	0	2	0	161	0	7	5	0	28	0	1	4	0	4	117	1	0	72	20

^a^Resistant (alive)

^b^Susceptible (dead)

^c^Total number of mosquitoes. The order of the genotypes is shown for loci 1016/1534/410. Resistant allele at locus 1016 = I, 1534 = C, 410 = L, triple-resistant genotype II/CC/LL, triple-susceptible genotype VV/FF/VV.

## Discussion

In Colombia, the use of pyrethroids for the control of *Ae*. *aegypti* is a fairly recent phenomenon. Among the pyrethroids, λ-cyhalothrin and deltamethrin have most commonly been used to control *Ae*. *aegypti* in Colombia. However, resistance to λ-cyhalothrin has been more commonly reported than resistance to deltamethrin in Colombia, as demonstrated by results from previous studies [7, 9–11, 13, 26] as well as those obtained in the present study. In the findings presented here, we detected resistance to permethrin and λ-cyhalothrin in all populations and varying degrees of susceptibility to deltamethrin. This heterogeneity of resistance patterns within the pyrethroid class suggests that diverse mechanisms are contributing to these phenotypes.

Resistance to DDT is widespread in Colombia owing to the application of this organochlorine compound for more than five decades in the country [20]. DDT and pyrethroids share the mode of action consisting of delayed sodium channel closure and membrane repolarization [41]. The modification of this target site due to the presence of *kdr* mutations on the *para* gene can lead to cross-resistance to both DDT and pyrethroids. As such, the high prevalence of *kdr* alleles detected in our study may also be linked to previous selection pressures caused by DDT [42, 43].

Our findings of resistance to λ-cyhalothrin in populations of *Ae*. *aegypti* in the Caribbean region of Colombia are consistent with those reported by Maestre *et al*. [13] for the populations of Barranquilla and Montería, Granada *et al*. [15] for the population of Riohacha, and Atencia *et al*. [31] for the population of Sincelejo. Likewise, Maestre *et al*. [13] reported resistance to λ-cyhalothrin in Valledupar and moderate resistance in Cartagena, while in the present work we detected resistance using the WHO bioassay but susceptibility using the CDC bioassay in both populations. However, other studies in Colombia have found resistance to λ-cyhalothrin using both the WHO and CDC techniques in the departments of Cundinamarca, Caquetá, Meta, Guaviare, Santander, Chocó, Antioquia, Putumayo and Casanare [9, 10, 12]. Ocampo *et al*. [11] found susceptibility to this insecticide using the CDC technique in the departments of Cauca, Nariño, Valle del Cauca and Huila.

Our findings of resistance to permethrin were consistent with those reported by Maestre *et al*. [13] for Barranquilla and Montería; however, for Cartagena and Valledupar, Maestre *et al*. had previously reported susceptibility, while we observed resistance using the WHO bioassay but susceptibility using the CDC bioassay. For this same insecticide Ardila *et al*. [12] encountered resistance in the department of Casanare, as did Fonseca e*t al*. [10] in the departments of Chocó Antioquia and Putumayo using both bioassay methodologies. The results of the intensity bioassays for both permethrin and λ-cyhalothrin for the populations that had shown resistance using the CDC methodology resulted in 100% mortality when the diagnostic dose was doubled, suggesting that while resistance was present in the populations, it had not yet reached a high level of intensity.

For deltamethrin, Maestre *et al*. [13] had reported resistance in Barranquilla; however, we found susceptibility using both bioassay methodologies. In Montería and Valledupar, Maestre *et al*. [13] reported resistance to deltamethrin in both populations, while we found susceptibility with the CDC bioassay and indications that resistance was developing with the WHO bioassay. In Cartagena, Maestre *et al*. [13] had reported moderate resistance to deltamethrin, while we observed resistance with the WHO bioassay and susceptibility with the CDC bioassay. Other studies carried out in Colombia showed resistance in *Ae*. *aegypti* to deltamethrin in the departments of Cundinamarca and Santander and susceptibility in the departments of Caquetá and Meta [9], as well as susceptibility in the departments of Cauca, Nariño, Valle del Cauca, Huila [11], Casanare [12] and Caldas [14] using the two bioassay methodologies.

In Colombia, both the CDC bioassay methodology and the WHO bioassay methodology have been used for insecticide susceptibility studies in adult *Ae*. *aegypti*. Typically, using both techniques, resistance to DDT has been observed in all *Ae*. *aegypti* populations evaluated in the country, together with variable susceptibility to pyrethroids and susceptibility to organophosphates in most populations [7]. In the present study, some discrepancies were observed between the results obtained with the WHO and CDC bioassay methodologies, indicating that the two techniques may not always provide consistent results. In studies by Aizoun *et al*. [44] and Fonseca *et al*. [20], WHO and CDC bioassays were compared to determine the susceptibility of *Anopheles gambiae* to deltamethrin and *Anopheles nuñeztovari* to fenitrothion. Both studies reported susceptibility when using the WHO bioassay and resistance when using the CDC bioassay. The authors observed that the exposure time of the mosquitoes to the insecticide (diagnostic time) was considerably shorter in the case of the CDC bioassay, which could have led to an overestimation of resistance; although in fact the opposite was observed in our study. Despite the shorter exposure time in the CDC bioassay, populations that were classified as resistant in the WHO bioassay were classified as susceptible in the CDC bioassay. This could potentially be explained due to the mechanisms underlying the resistance; for example, resistance that is primarily caused by *kdr* would likely result in populations that are not quickly knocked down and thus scored as ‘resistant’ at 30 minutes. However, if the main mechanisms of resistance are metabolic, mosquitoes may initially be knocked down but could recover over time as their detoxification enzymes metabolize the insecticide. Indeed, our biochemical assay data suggest that elevated enzymatic activity is present in the populations that were studied.

Most previous studies regarding enzymatic activity have been conducted on *Ae*. *aegypti* populations from other regions of Colombia where alterations were detected, mainly in MFOs and nonspecific esterases, in populations from Antioquia, Chocó, Putumayo, Cauca, Valle del Cauca, Nariño, Huila, Santander, Meta, and Casanare [9–12]. The one previous study conducted in the Caribbean region of Colombia reported altered α-esterases and MFOs in *Ae*. *aegypti* from Valledupar, MFOs in Cienaga, and GSTs in Sincelejo. In that previous study, no alterations in enzyme activity were detected in Cartagena, Montería, Barranquilla, San Juan, Puerto Colombia, and Soledad, [13]. Our results are consistent with the finding of highly altered MFOs in Valledupar, and we also detected altered β-esterases in that same population. We also detected highly altered α-esterases, β-esterases, MFOs and GSTs in Montería; altered β-esterases and GSTs in Barranquilla; and altered GSTs in Cartagena. Additionally, in the present study we detected altered pNPA-esterases in the population of Juan de Acosta.

Regarding esterases, studies to date have reported the overexpression of β-esterases in populations resistant to organophosphates and pyrethroids [9–11]. Altered levels of α-esterase activity were detected previously in Valledupar in the study conducted by Maestre *et al*. [13]. In other countries, altered α-esterases, β-esterases, and MFOs have been reported in *Ae*. *aegypti* populations resistant to organophosphates, carbamates, and pyrethroids [40, 45–51].

There are no studies in Colombia incriminating insensitive acetylcholinesterase as a mechanism associated with resistance to organophosphates and carbamates in *Ae*. *aegypti*. A study by Grisales *et al*. [24] reported resistance to temephos in the population of *Ae*. *aegypti* from Cúcuta (RR: 15X) without evidence of insensitive acetylcholinesterase, although they did detect esterase and oxidase-based mechanisms.

*Kdr* mutations are important mechanisms involved in DDT and pyrethroid resistance. In Colombia, the first *kdr* mutation reported in populations of *Ae*. *aegypti* was V1016I, which was identified in populations from Puerto Colombia, Soledad, Barranquilla, Valledupar, San Juan, Sincelejo, Montería, Cienaga and Cartagena, which are all located in the Caribbean region. In that initial report, the V1016I mutation showed frequencies ranging between 0.07 and 0.35; the lowest frequency was found in the Cienaga population and the highest was found in Soledad, Montería, and Barranquilla, with frequencies of 0.35, 0.33, and 0.32, respectively [13]. The highest frequency of 1016I that we detected in the present study was in Montería, with a frequency of 0.70, showing a large increase in the frequency in this population from what was originally reported by Maestre *et al*. [13]. In addition, an increase in the frequency of 1016I from 0.09 to 0.16 was detected in Cartagena and a reduced frequency was detected in Barranquilla and Valledupar, from 0.32 and 0.27, respectively, to 0.15 in both populations. V1016I had also previously been reported in Quindío at low levels of frequency (0.02–0.05) [25].

The F1534C mutation was first detected in Colombia in the department of Sincelejo (Sucre), in the Caribbean region [31]. It had also previously been reported in *Ae*. *aegypti* populations from Puerto Colombia, Soledad, Barranquilla, Valledupar, San Juan, Sincelejo, Montería, Cienaga and Cartagena with frequencies ranging between 0.74 and 0.88. When compared with the results reported previously, we observed increased frequencies of 1534C, having risen in Barranquilla from 0.74 to 0.76, in Cartagena from 0.86 to 0.97, in Montería from 0.88 to 1.00, and in Valledupar from 0.82 to 0.94. These increases are likely attributable to the constant pressure exerted by pyrethroid insecticides, which were heavily applied during the period between the two studies for the control of dengue, chikungunya, and Zika. Although there are no previous studies reporting this mutation in Juan de Acosta and Chiriguaná, these populations also showed high frequencies (0.76 and 0.95, respectively). Moreover, high frequencies of 1534C have been reported in other areas of Colombia, including Villavicencio, Riohacha, and Bello, with frequencies of 0.63, 0.71, and 0.56, respectively [15]. In these latter three populations, the V410L mutation was also identified in Colombia for the first time, with frequencies of 0.46, 0.30, and 0.06, respectively. It is noteworthy that in that study, *Ae*. *aegypti* from Bello were susceptible to λ-cyhalothrin, whereas those from Riohacha and Villavicencio were resistant. In these latter two populations, the researchers detected a positive association between V410L and V1016I and resistance to λ-cyhalothrin. In the present study, the V410L mutation was detected for the first time in the study populations, with frequencies ranging between 0.05 in Valledupar and 0.72 in Montería. The frequencies of the V1016I mutation were very similar to those of the V410L mutation in all the evaluated populations; this result is consistent with the findings reported by Granada *et al*. [15] for *Ae*. *aegypti* in Bello, Villavicencio, and Riohacha.

Haddi *et al*. [39] reported the presence of the V410L mutation in resistant *Ae*. *aegypti* in Brazil and observed that this mutation, either alone or in combination with the F1534C mutation, was strongly associated with increased the resistance to type I and II pyrethroids. This is consistent with the results of the present study, where the 1534C and 410L alleles were associated with resistance to permethrin in the population of Juan de Acosta. The 1016I, 1534C, and 410L alleles were all associated with resistance to permethrin in the Chiriguaná, Montería, and Valledupar populations based on phenotyping by the WHO bioassay. In addition, F1534C was associated with resistance to deltamethrin in Chiriguaná, Valledupar, and Montería; V1016I and V410L were also associated with deltamethrin resistance in the case of the latter population. Similarly, an association was found between all three mutations and resistance to λ-cyhalothrin in Valledupar, Montería, and Juan de Acosta. This last result is consistent with the results of the study by Maestre *et al*. [32] which detected a significant positive correlation between the frequency of the 1016I allele and resistance to permethrin, λ-cyhalothrin, and cyfluthrin. However, no significant correlation was observed in that same study between 1534C and resistance to any pyrethroids [32].

Recent studies conducted in Mexico proposed three sequential models to explain the evolution of the V1016I, F1534C, and V410L mutations. The first model suggests that F1534C appeared first, providing low resistance levels, followed by the appearance of V1016I, which provided higher levels of resistance. The second model challenges the first model and proposes that V410L and V1016I occurred independently on a C1534 haplotype followed by cis conversion by recombination. Finally, a third model assumes that the three mutations appeared independently at low frequencies and that two recombination events rearranged them in a cis configuration [52]. Considering these previous models and the results obtained in the present investigation, it is possible to hypothesize that the appearance of V410L and V1016I did not occur independently because their allelic frequencies were so similar and they almost always appeared together.

Regarding the 1016/1534/410 phenotype–genotype association, a relationship between the VI_1016_/CC_1534_/VL_410_ genotype and resistance to λ-cyhalothrin and permethrin was detected in the present study. These results are consistent with the study conducted by Haddi *et al*. [39] in a pyrethroid-resistant *Ae*. *aegypti* strain from Brazil, where V410L alone or in combination with F1534C was shown to reduce sodium channel sensitivity to type I (permethrin) and type II pyrethroids (λ-cyhalothrin and deltamethrin). In addition, these results further support the notion that the presence of VI_1016_ and VL_410_ heterozygotes is sufficient to confer resistance to deltamethrin [52]. These findings suggest that the interactions of multiple mutations play a role in the response of *Ae*. *aegypti* sodium channels to insecticides [53].

In Colombia, previous studies have identified the frequency of V1016I, F1534C and V410L in populations of *Ae*. *aegypti* and have correlated those frequencies with the results of outcomes obtained through the CDC bioassay technique for populations from Puerto Colombia, Soledad and Barranquilla (Atlántico); Valledupar and San Juan (Cesar); Sincelejo (Sucre); Montería (Córdoba); Cienaga (Magdalena); Cartagena (Bolívar) [32]; Villavicencio (Meta); Riohacha (La Guajira); Bello (Antioquia) [15]; Giron (Santander); Buga, Palmira, Yumbo, Cali (Valle del Cauca) and Medellin (Antioquia) [54]. Unlike the aforementioned studies, our study evaluated for the first time for Colombia the direct association between phenotype and genotype in individual mosquitoes phenotyped through CDC and WHO bioassays. Furthermore, the present study established for the first time for Colombia differences between the phenotypes observed in CDC and WHO bioassays for pyrethroids and trilocus *kdr* haplotypes present in populations of *Ae*. *aegypti* from Montería (Cordoba); Cartagena (Bolívar); Juan de Acosta, Barranquilla (Atlántico); Chiriguaná and Valledupar (Cesar). This contributes to knowledge about the role and co-occurrence of these mutations in *Ae*. *aegypti* from the Caribbean region and how they relate to phenotypic resistance, providing further evidence to guide the selection of insecticides to be used in *Ae*. *aegypti* control.

A key strength of the present study is that it expands the knowledge base regarding the susceptibility status of *Ae*. *aegypti* to pyrethroid insecticides in Colombia. Additionally, it provides new information regarding the frequency and distribution of *kdr* mutations and a detailed analysis of phenotype-genotype associations. An increased understanding of the role of the mechanisms involved in resistance will contribute to improved resistance surveillance strategies which can better guide control programs for the selection of insecticides for the control of *Ae*. *aegypti*. An important limitation of the current study is the lack of synergist bioassays, which would permit an estimation of the relative contribution of metabolic mechanisms as compared to *kdr* in conferring phenotypic resistance.

Regardless, the results presented here provide important input for territorial and national entities in vector control decision-making. In addition, the detailed information on resistance mechanisms provide deeper insight into the types of resistance mitigation and management strategies might be most effective in the populations evaluated.

## Conclusions

Variability was observed in pyrethroid susceptibility using the WHO and CDC bioassay methodologies, highlighting the importance of using a consistent methodology to routinely screen populations for susceptibility. The altered activity levels of β-esterases, α-esterases, MFOs, and GSTs suggest that metabolic resistance may be important in these populations. The *kdr* mutations V1016I, F1534C, and V410L were detected in all populations, with 1534C being nearly fixed in all except two populations. Finally, associations were observed between the F1534C mutation and resistance to permethrin in all populations, the F1534C mutation with resistance to deltamethrin in Chiriguaná, Montería, and Valledupar, and the V1016I, F1534C, and V410L mutations and resistance to λ-cyhalothrin in Juan de Acosta, Valledupar, and Montería.

## Supporting information

S1 Table Mortality of populations of *Ae*. *aegypti* evaluated against diagnostic concentrations of pyrethroid insecticides following WHO methodology. (XLSX)

S2 Table Mortality of populations of *Ae*. *aegypti* evaluated against diagnostic doses of pyrethroid insecticides following CDC methodology. (XLSX)

S3 Table Enzymatic activity levels *Aedes aegypti* populations the study and Rockefeller strain. a: insensitive acetylcholinesterase, b: Total protein, c: glutathione-S-transferases, d: α-esterases, e: β-esterases, f: pNPA- esterases, g: mixed-function oxidases.
(XLSX)

S4 Table Summary of frequencies of the 13 tri-locus genotypes present in F0 *Ae*. *aegypti* females. The order of the genotypes is 1016/1534/410. Mutant alleles: 1016 = I, 1534 = C, and 410 = L.
(XLSX)

## References

[1] Padilla JC, Lizarazo FE, Murillo OL, Mendigaña FA, Pachón E, Vera MJ. Epidemiología de las principales enfermedades transmitidas por vectores en Colombia, 1990–2016. Biomedica. 2017;37(Supl.2):27–40. 29165933 10.7705/biomedica.v37i0.3769

[2] World Health Organization. Dengue y dengue grave [Internet]. [cited 2018 Apr 30]. Available from: https://www.who.int/es/news-room/fact-sheets/detail/dengue-and-severe-dengue

[3] Instituto Nacional de Salud. Informe de evento. Dengue: Colombia, 2017 [Internet]. [cited 2018 May 20]. Available from: https://www.ins.gov.co/buscador-eventos/Informesdeevento/DENGUE2017.pdf

[4] Instituto Nacional de Salud. Sistema de Vigilancia Epidemiologico Nacional: Enfermedades Transmitidas por Vectores [Internet]. [cited 2018 May 23]. Available from: http://portalsivigila.ins.gov.co/sivigila/documentos/Docs_1.php

[5] Tovar-Sanchez Z, Bolívar_pertuz S, Maestre-Serrano R. Chikungunya: aspectos generales de una enfermedad emergente en Colombia Chikungunya: general aspects of an emerging disease in Colombia. Rev Biociencias. 2015;10(1):75–88.

[6] Instituto Nacional de Salud. Informe de evento: Enfermedad por virus Zika. Colombia, 2017 [Internet]. [cited 2018 May 23]. Available from: http://www.ins.gov.co/buscador-eventos/Informesdeevento/ZIKA2017.pdf

[7] Maestre-Serrano R. Susceptibility status of *Aedes aegypti* to insecticides in Colombia. In: Insecticides–Pest Engineering [Internet]. 2012. p. 163–200. Available from: http://www.scielo.org.co/scielo.php?script=sci_arttext&pid=S0120-04882010000200012&lng=en&nrm=iso&tlng=es

[8] Maestre-Serrano R, Pacheco-Lugo L, Salcedo-Mendoza S. Índices de infestación aédica e identificación de conocimientos, actitudes y prácticas sobre dengue en llanterías del Departamento del Atlántico, Colombia. (Spanish). Rev Salud Pública [Internet]. 2015;17(5):738–48. Available from: http://10.0.60.86/rsap.v17n5.3534%5Cnhttp://search.ebscohost.com/login.aspx?direct=true&db=lth&AN=113247085&lang=es&site=ehost-live 10.15446/rsap.v17n5.35345 28453051

[9] Santacoloma L, Chaves B, Brochero H. Susceptibilidad de *Aedes aegypti* a DDT, deltametrina y lambdacialotrina en Colombia. Rev Panam Salud Publica/Pan Am J Public Heal. 2010;27(1):66–73. 10.1590/s1020-49892010000100010 20209234

[10] Fonseca-González I, Quiñones ML, Lenhart A, Brogdon WG. Insecticide resistance status of *Aedes aegypti* (L.) from Colombia. Pest Manag Sci. 2011;67(4):430–7. 10.1002/ps.2081 21394876

[11] Ocampo CB, Salazar-Terreros MJ, Mina NJ, McAllister J, Brogdon W. Insecticide resistance status of *Aedes aegypti* in 10 localities in Colombia. Acta Trop [Internet]. 2011;118(1):37–44. Available from: 10.1016/j.actatropica.2011.01.007 21300017

[12] Ardila-Roldán S, Santacoloma L, Brochero H. Estado de la sensibilidad a los insecticidas de uso en salud pública en poblaciones naturales de *Aedes aegypti* (Diptera: Culicidae) del departamento de Casanare, Colombia. Biomedica. 2013;33(3):446–58. 10.7705/biomedica.v33i3.1534 24652181

[13] Maestre-Serrano R, Gomez-Camargo D, Ponce-Garcia G, Flores AE. Susceptibility to insecticides and resistance mechanisms in *Aedes aegypti* from the Colombian Caribbean Region. Pestic Biochem Physiol [Internet]. 2014;116:63–73. Available from: 10.1016/j.pestbp.2014.09.014 25454522

[14] Conde M, Orjuela LI, Castellanos CA, Herrera-Varela M, Licastro S, Quiñones ML. Evaluación de la sensibilidad a insecticidas en poblaciones de *Aedes aegypti* (Diptera: Culicidae) del departamento de Caldas, Colombia, en 2007 y 2011. Biomedica. 2015;35(1):43–52. 10.1590/S0120-41572015000100007 26148033

[15] Granada Y, Mar A, Strode C, Triana-chavez O. A Point Mutation V419L in the Sodium Channel Gene from Natural Populations of *Aedes aegypti* Is Involved in Resistance to λ -Cyhalothrin in Colombia. Insects. 2018;9:23–35. 10.3390/insects9010023 PMC5872288 29443870

[16] Bisset LJ a. Uso correcto de insecticidas: control de la resistencia. Rev Cubana Med Trop. 2002;54(3):202–19. 15846946

[17] Du Y, Nomura Y, Zhorov BS, Dong K. Sodium channel mutations and pyrethroid resistance in *Aedes aegypti*. Insects. 2016;7(4):60–71. 10.3390/insects7040060 PMC5198208 27809228

[18] Salazar M, Carvajal A, Cuellar ME, Olaya A, Quiñones J, Velasquez OL, et al Resistance to insecticides in populations of *Aedes aegypti* and *Anopheles spp*. in the departments of Huila, Valle Cauca and Nariño. In: Biomedica, editor. XIII Congreso colombiano de parasitologia y medicina tropical. Bogota; 2007 p. 177.

[19] Maestre RS, Rey G V., De Las J, Vergara CS, Santacoloma L V., Goenaga SO, et al Susceptibilidad de *Aedes aegypti* (Diptera: Culicidae) a temefos en Atlantico-Colombia. Rev Colomb Entomol. 2009;35(2):202–5.

[20] Fonseca-González I BD. Variación temporal en la susceptibilidad a malatión y lambdacialotrina en *Aedes aegypti* (L) de Quibdó, Colombia. In: Biomedica, editor. Congreso de Parasitologia y Medicina Tropical. Ibague; 2009 p. 216–34.

[21] Maestre-Serrano R, Florez Z, Cabrera C, Goenaga S, Gomez D GC. Susceptibility status of *Aedes aegypti* to insecticides in la Guajira (colombia). In: Hygiene TAJ of TM and, editor. The American Journal of Tropical Medicine and Hygiene. Atlanta, Georgia; 2010 p. 99–141.

[22] Maestre-Serrano R, Rey Gabriela, De las salas J, Vergara C, Santacoloma L, Goenaga S, et al Estado de la susceptibilidad de *Aedes aegypti* a insecticidas en Atlántico (Colombia). Rev Colomb Entomol. 2010;36(2):242–8.

[23] Santacoloma L, Chaves B, Brochero HL. Estado de la susceptibilidad de poblaciones naturales del vector del dengue a insecticidas en trece localidades de Colombia. Biomedica. 2012;32(3):333–43. 10.1590/S0120-41572012000300004 23715182

[24] Grisales N, Poupardin R, Gomez S, Fonseca-Gonzalez I, Ranson H, Lenhart A. Temephos Resistance in *Aedes aegypti* in Colombia Compromises Dengue Vector Control. PLoS Negl Trop Dis. 2013;7(9). 10.1371/journal.pntd.0002438 PMC3777894 24069492

[25] Aguirre-Obando OA, Dalla Bona AC, Duque L JE, Navarro-Silva MA. Insecticide resistance and genetic variability in natural populations of Aedes (*Stegomyia*) *aegypti* (Diptera: Culicidae) from Colombia. Zoologia. 2015;32(1):14–22.

[26] Instituto Nacional de Salud. Red de vigilancia de la resistencia a insecticidas de uso en salud pública en Colombia, año 2018 [Internet]. [cited 2018 May 20]. Available from: http://www.ins.gov.co/buscador-eventos/Informacindelaboratorio/Informe-VRI-2018.pdf

[27] Suárez MF, González R MC. Temefos resistance to *Aedes aegypti* in Cali, Colombia. In: Am J Trop Med Hyg, editor. 45th Annual meeting of the American Society of Tropical Medicine and Hygiene. Baltimore, Maryland; 1996. p. 257.

[28] Rojas W, González J, Amud M, Quiñones M VI. Evaluation of the susceptibility of *Aedes aegypti* of the municipality of Barrancabermeja, Santander, to the insecticides malathion, fenitrothion, temephos, lambda-cyhalothrin, deltamethrin, permethrin, pro. In: Biomedica, editor. Congreso colombiano de medicina tropical. 2003. p. 56.

[29] Anaya Y, Cochero S, Rey G S l. Assessment of susceptibility to insecticides of *Aedes aegypti* caught in Sincelejo. In: Biomedica, editor. XIII Congreso colombiano de parasitologia y medicina tropical. Bogota; 2007. p. 257.

[30] Aguirre-Obando OA, Martins AJ, Navarro-Silva MA. First report of the Phe1534Cys kdr mutation in natural populations of *Aedes albopictus* from Brazil. Parasites and Vectors. 2017;10(1):160–70. 10.1186/s13071-017-2089-5 28347326 PMC5369189

[31] Atencia MC, Pérez M de J, Jaramillo MC, Caldera SM, Cochero S, Bejarano EE. First report of the F1534C mutation associated with cross-resistance to DDT and pyrethroids in *Aedes aegypti* from Colombia. Biomedica. 2016;36(3):432–7. 10.7705/biomedica.v36i3.2834 27869391

[32] Maestre-Serrano R, Pareja-Loaiza P, Gomez Camargo D, Ponce-García G, Flores AE. Co-occurrence of V1016I and F1534C mutations in the voltage-gated sodium channel and resistance to pyrethroids in *Aedes aegypti* (L.) from the Colombian Caribbean region. Pest Manag Sci. 2019;75(6):1681–8. 10.1002/ps.5287 30520256

[33] Brogdon WG, McAllister JC. Insecticide resistance and vector control. Emerg Infect Dis. 1998;9(2):605–13. 10.3201/eid0404.980410 PMC2640263 9866736

[34] World Health Organization—WHO. Test procedures for insecticide resistance monitoring in malaria vector mosquitoes. Second. Ginebra; 2017. 1–50 p.

[35] Valle D, Montella IR. Quantification methodology for enzyme activity related to insecticide resistance in *Aedes aegypti*. Vol. 1, Brasil. Ministério da Saúde. Fundação Oswaldo Cruz. 2006. 128 p.

[36] Brogdon WG. Mosquito protein microassay-I. Protein determinations from small portions of single-mosquito homogenates. Comp Biochem Physiol—Part B Biochem. 1984;79B(3):457–9. 10.1016/0305-0491(84)90405-x 6509934

[37] Saavedra-Rodriguez K, Urdaneta-Marquez L, Rajatileka S, Moulton M, Flores AE, Fernandez-Salas I, et al A mutation in the voltage-gated sodium channel gene associated with pyrethroid resistance in Latin American *Aedes aegypti*. Insect Mol Biol. 2007;16(6):785–98. 10.1111/j.1365-2583.2007.00774.x 18093007

[38] Yanola J, Somboon P, Walton C, Nachaiwieng W, Somwang P, Prapanthadara L aied. Analyses à haut débit pour la détection de la mutation F1534C dans le gène du canal sodium voltage dépendant d’Aedes aegypti résistant au perméthrine et distribution de cette mutation à travers la Thaïlande. Trop Med Int Heal. 2011;16(4):501–9. 10.1111/j.1365-3156.2011.02725.x 21342372

[39] Haddi K, Tomé HVV, Du Y, Valbon WR, Nomura Y, Martins GF, et al Detection of a new pyrethroid resistance mutation (V410L) in the sodium channel of *Aedes aegypti*: A potential challenge for mosquito control. Sci Rep [Internet]. 2017;7(March):1–9. Available from: 10.1038/srep46549 PMC5396194 28422157

[40] Montella IR, Martins AJ, Viana-Medeiros PF, Lima JBP, Braga IA, Valle D. Insecticide resistance mechanisms of Brazilian *Aedes aegypti* populations from 2001 to 2004. Am J Trop Med Hyg. 2007;77(3):467–77. 17827362

[41] Zlotkin E, Devonshire AL, Warmke JW. The pharmacological flexibility of the insect voltage gated sodium channel: Toxicity of AaIT to knockdown resistant (*kdr*) flies. Insect Biochem Mol Biol. 1999;29(10):849–53. 10.1016/s0965-1748(99)00079-x 10528405

[42] Rodríguez MM, Bisset J, Ruiz M, Soca A. Cross-Resistance to Pyrethroid and Organophosphorus Insecticides Induced by Selection with Temephos in *Aedes aegypti* (Diptera: Culicidae) from Cuba. J Med Entomol. 2002;39(6):882–8. 10.1603/0022-2585-39.6.882 12495187

[43] Tikar SN, Kumar A, Prasad GBKS, Prakash S. Temephos-induced resistance in *Aedes aegypti* and its cross-resistance studies to certain insecticides from India. Parasitol Res. 2009;105(1):57–63. 10.1007/s00436-009-1362-8 19229558

[44] Aïzoun N, Ossè R, Azondekon R, Alia R, Oussou O, Gnanguenon V, et al Comparison of the standard WHO susceptibility tests and the CDC bottle bioassay for the determination of insecticide susceptibility in malaria vectors and their correlation with biochemical and molecular biology assays in Benin, West Africa. Parasites and Vectors. 2013;6(1):147–56. 23688233 10.1186/1756-3305-6-147 PMC3669035

[45] Rodríguez M, BIsset M, Ditter F, Omayda P. Resistencia a insecticidas en larvas y adultos de *Aedes aegypti*: prevalencia de la esterasa A4 asociada con la resistencia a temefos. Rev Cubana Med Trop. 2004;56(1):54–60. 15849910

[46] Flores AE, Albeldaño-Vázquez W, Salas IF, Badii MH, Becerra HL, Garcia GP, et al Elevated α-esterase levels associated with permethrin tolerance in *Aedes aegypti* (L.) from Baja California, Mexico. Pestic Biochem Physiol. 2005;82(1):66–78.

[47] Flores AE, Grajales JS, Salas IF, Garcia GP, Becerra HL, Lozano S, et al Mechanisms of Insecticide Resistance in Field Populations of *Aedes aegypti* (L.) From Mechanisms of Insecticide Resistance in Field Populations. 2006;22(4):672–7. 10.2987/8756-971X(2006)22[672:MOIRIF]2.0.CO;2 17304936

[48] Harris AF, Rajatileka S, Ranson H. Pyrethroid resistance in *Aedes aegypti* from Grand Cayman. Am J Trop Med Hyg. 2010;83(2):277–84. 10.4269/ajtmh.2010.09-0623 20682868 PMC2911171

[49] Rodríguez MM, Bisset JA, Ricardo Y, Pérez O, Montada D, Figueredo D, et al Resistencia a insecticidas organofosforados en Aedes aegypti (Diptera: Culicidae) de Santiago de Cuba, 1997–2009. Rev Cubana Med Trop [Internet]. 2010;62(3):217–23. Available from: http://scielo.sld.cu/scielo.php?script=sci_arttext&pid=S0375-07602010000300009&lng=es&nrm=iso&tlng=es 23437552

[50] Polson KA, Brogdon WG, Rawlins SC, Chadee DD. Characterization of insecticide resistance in Trinidadian strains of *Aedes aegypti* mosquitoes. Acta Trop [Internet]. 2011;117(1):31–8. Available from: 10.1016/j.actatropica.2010.09.005 20858454

[51] Alvarez LC, Ponce G, Oviedo M, Lopez B, Flores AE. Resistance to Malathion and Deltamethrin in *Aedes aegypti* (Diptera: Culicidae) From Western Venezuela. J Med Entomol. 2013;50(5):1031–9. 10.1603/me12254 24180108

[52] Saavedra-Rodriguez K, Maloof FV, Campbell CL, Garcia-Rejon J, Lenhart A, Penilla P, et al Parallel evolution of vgsc mutations at domains IS6, IIS6 and IIIS6 in pyrethroid resistant *Aedes aegypti* from Mexico. Sci Rep. 2018;8(1):6749–55. 10.1038/s41598-018-24642-2 29712956 PMC5928250

[53] Liu N. Insecticide Resistance in Mosquitoes: Impact, Mechanisms, and Research Directions. Annu Rev Entomol. 2015;60(1):537–59. 25564745 10.1146/annurev-ento-010814-020828

[54] Angélica Aponte, R. Patricia Penilla, Rodríguez Américo D.and CBO. Mechanisms of Pyrethroid Resistance in *Aedes* (*Stegomyia*) *aegypti* from Colombia. Acta Trop. 2019;191(1):146–54. 30552882 10.1016/j.actatropica.2018.12.021 PMC6447284

